# Construction of an evaluation indicator system for humanistic care quality in nursing homes

**DOI:** 10.1186/s12877-026-07623-3

**Published:** 2026-05-08

**Authors:** Chenglei Wu, Sisi Zhang, Huiqi Chen, Jing Yu, Jingru Song, Yanan Shi, Qin Shen

**Affiliations:** School of Nursing, Zhejiang Chinese Medical University, Hangzhou, 310053 China

**Keywords:** Nursing homes, Humanistic care, Analytic hierarchy process (AHP), Delphi Method, Quality indicators

## Abstract

**Background:**

The implementation of humanistic care services in nursing homes is of great importance for improving the quality of life of older people, enhancing their sense of self-worth, and meeting their spiritual and psychological needs. However, there is currently a lack of standardized criteria and validated tools for the systematic assessment of the quality of humanistic care in nursing homes. Therefore, the aim of this study was to develop a comprehensive indicator system for evaluating the quality of humanistic care in nursing homes.

**Methods:**

Guided by the Quality Caring Model and Maslow’s hierarchy of needs theory as the theoretical framework, an initial evaluation indicator system was developed through a literature review and semi-structured interviews. Based on the preliminary indicator system, an expert consultation questionnaire was designed, and two rounds of Delphi expert consultation were conducted with 32 experts from relevant fields to further refine and optimize the evaluation indicator system by integrating expert opinions. Subsequently, the Analytic Hierarchy Process was applied to determine the weight of each indicator, and consistency testing was performed.

**Results:**

The effective recovery rates of the two rounds of expert consultation questionnaires were 94.12% and 100.00%, respectively, and the expert authority coefficients were 0.850 and 0.859. The Kendall’s coefficients of concordance for the two rounds of expert consultation were 0.167 and 0.269, respectively (*P* < 0.001). Ultimately, a quality evaluation indicator system for humanistic care in nursing homes was established, comprising 3 primary-level indicators, 10 secondary-level indicators, and 38 tertiary-level indicators.

**Conclusion:**

The quality evaluation indicator system for humanistic care in nursing homes developed in this study demonstrates a certain degree of scientific rigor and rationality. It can provide a theoretical reference for promoting the standardization of humanistic care processes and the systematization of quality management in nursing homes in China, and offers a systematic framework for the subsequent evaluation and improvement of the quality of humanistic care.

**Supplementary Information:**

The online version contains supplementary material available at 10.1186/s12877-026-07623-3.

## Background

Global population aging has become a common challenge faced by countries worldwide [1]. In this context, population aging in China is particularly severe, as the country not only has the largest older population in the world but is also experiencing one of the fastest rates of population aging globally [2]. According to data from the Seventh National Population Census released by the National Bureau of Statistics of China [3], as of 2020, people aged 60 years and above and those aged 65 years and above accounted for 18.70% and 13.50% of the total population in China, respectively. Compared with the Sixth National Population Census, the proportions of the population in these two age groups increased by 5.44% and 4.63%, respectively. It is projected that the size of the older population in China will reach a peak of 437 million by 2051 [4]. In this context, China has gradually developed a care system for older people that is centered on home-based care, supported by community-based services, and underpinned by nursing homes.

However, family structures in China are undergoing significant transformation, with nuclear families increasingly becoming predominant [5], resulting in a serious shortage of caregiving resources for long-term home-based care for older people and making it difficult to meet their care needs [6, 7]. At the same time, community-based care has not yet been fully developed and continues to face challenges such as insufficient service provision and a shortage of qualified professionals [8, 9]. Within this context, institutional care, owing to its advantages in service professionalization, has gained widespread public recognition and has gradually become a primary care option for older people [10].

After admission to nursing homes, older people often experience loneliness, difficulties in adjustment, and depressive symptoms due to unfamiliar environments, the need to rebuild social networks, and declines in physical functioning [11–13]. Humanistic care in nursing homes refers to a value-oriented approach to care that emphasizes respect for the dignity, emotional needs, and individuality of older people, and aims to provide person-centered care through supportive interpersonal relationships and a caring environment [14, 15]. Existing studies have demonstrated that humanistic care has positive effects on the physical and mental health of older people. Ballard et al. found that the implementation of humanistic care interventions for people with dementia in nursing homes improved their quality of life while reducing agitation and neuropsychiatric symptoms [16]. Similarly, Li et al. reported that humanistic care practices in nursing homes were associated with improvements in residents’ psychological well-being and significantly enhanced nursing care satisfaction [17]. Consistent with the aforementioned evidence, policy authorities and professional organizations in China have also emphasized, at the macro level, the development of mental health and humanistic care services in nursing homes. *The Healthy China 2030 Plan* calls for the active development of mental health and humanistic care services for older people in nursing homes [18]. Similarly, the Chinese Association of Social Security has indicated that, in advancing the healthy development of the elderly care service sector, it is essential to give sustained attention to humanistic care, strengthen its implementation, and integrate its underlying values into the institutional framework of elderly care services [19].

However, due to limitations in management and supervision systems, the quality of humanistic care in nursing homes across China shows substantial variation, leading to a range of practical issues. Studies have indicated that many nursing homes continue to focus primarily on meeting older people’s basic daily care needs, while paying insufficient attention to their psychological needs, emotional support, and individual dignity, resulting in humanistic care services that lack both systematic structure and personalization [20]. In addition, with regard to the institutional care environment, some nursing homes have not fully implemented a person-centered care philosophy, and deficiencies remain in areas such as comfort, privacy protection, and safety [21, 22]. Therefore, to safeguard the quality of later life for older people and to enhance their satisfaction with humanistic care, it is essential to strengthen the management and regulation of the quality of humanistic care in nursing homes.

*The 14th Five-Year Plan for the Development of National Aging Programs and the Elderly Care Service System* explicitly proposes accelerating the establishment of a nationally unified quality standard system for elderly care services [23], thereby providing clear guidance for quality management. In this context, how to scientifically evaluate the quality of services in nursing homes—particularly the quality of humanistic care—has become a central issue in improving the overall standard of elderly care services. However, existing research and practice still exhibit clear limitations. In China, studies on quality evaluation in elderly care institutions have primarily focused on aspects such as nursing care quality, assessment of older people’s service needs, core competencies of nursing staff, or safety management [24–28], while relatively few studies have examined evaluation tools specifically targeting the quality of humanistic care. Most existing evaluation tools adopt a single-dimensional approach and focus primarily on assessing humanistic care processes, while evaluation indicators related to structural and outcome aspects remain relatively scarce. As a result, their scope of coverage is limited, and an integrated, coherent evaluation framework with internally connected components has yet to be established. In addition, existing evaluation indicator systems for the quality of humanistic care in nursing homes developed in Taiwan are difficult to be directly applied to mainland China due to differences in levels of economic development, elderly care service models, and cultural contexts [29].

In the practice of humanistic care, without systematic management and institutional support, reliance solely on the personal qualities and professional ethics of nursing staff often makes it difficult to achieve sustained and stable development at the institutional level. Therefore, humanistic care requires guidance and assurance through a scientific and standardized quality evaluation system. Quality evaluation runs throughout the entire process of management activities and serves as an essential means by which management organizations, based on relevant standards, systematically examine and analyze service processes and outcomes, thereby objectively identifying quality variations and promoting continuous improvement. Establishing a scientific quality evaluation system for humanistic care facilitates the monitoring of management performance and provides a basis for standardizing service practices and evaluating implementation effectiveness [30]. Therefore, this study aimed to scientifically develop a comprehensive, concise, and operational evaluation indicator system for the quality of humanistic care in nursing homes in China, in order to provide a scientific basis for the standardized implementation of humanistic care services and the continuous improvement of care quality in nursing homes.

## Methods

**Fig. 1:**
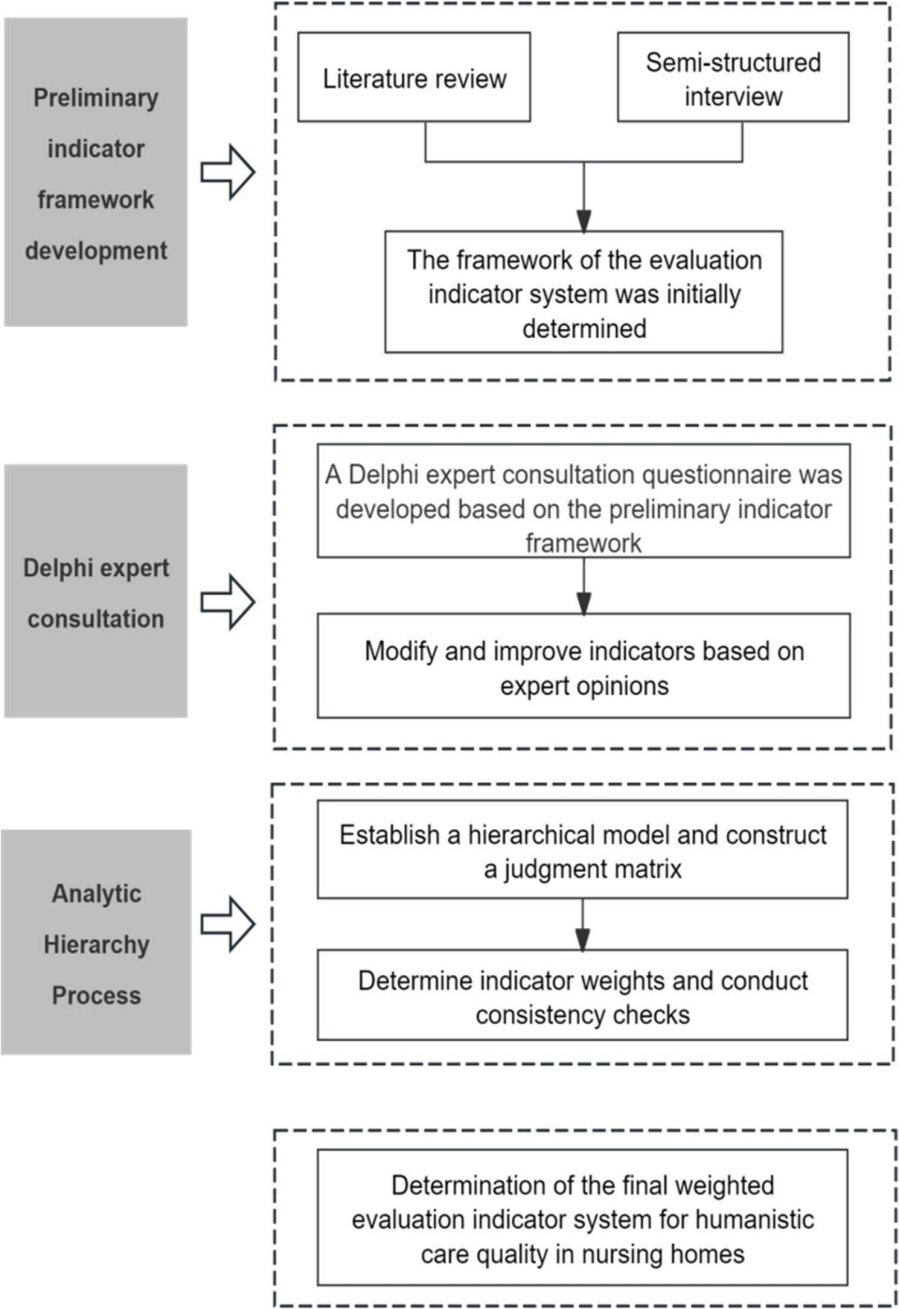
Flowchart of the development and weighting process of the evaluation indicator system

Based on the Quality Caring Model and Maslow’s hierarchy of needs theory, an evaluation indicator system for humanistic care quality in nursing homes was developed through a literature review, semi-structured interviews, Delphi expert consultation, and the Analytic Hierarchy Process (AHP). Specifically, the literature review and semi-structured interviews were used to develop the preliminary indicator framework, Delphi expert consultation was used to modify and improve the indicators, and AHP was subsequently applied to determine indicator weights and conduct consistency checks. The flowchart of the development and weighting process of the evaluation indicator system is presented in Fig. 1.

### Establish a research team

The research team consisted of nine members: one professor specializing in geriatric nursing, one professor in nursing management, one associate professor in public health, one director of a nursing home, one director of a nursing department, one head nurse, one nursing professional with more than 10 years of experience in nursing home care, and two master’s students in nursing whose research focus was geriatric nursing. The team was responsible for selecting the theoretical framework, conducting the literature review, carrying out semi-structured interviews, drafting the preliminary indicator system, inviting experts for the Delphi consultation, summarizing and analyzing feedback from each round of expert consultation, and determining the weight coefficients of the indicators.

### Selection of the theoretical framework

**Fig. 2:**
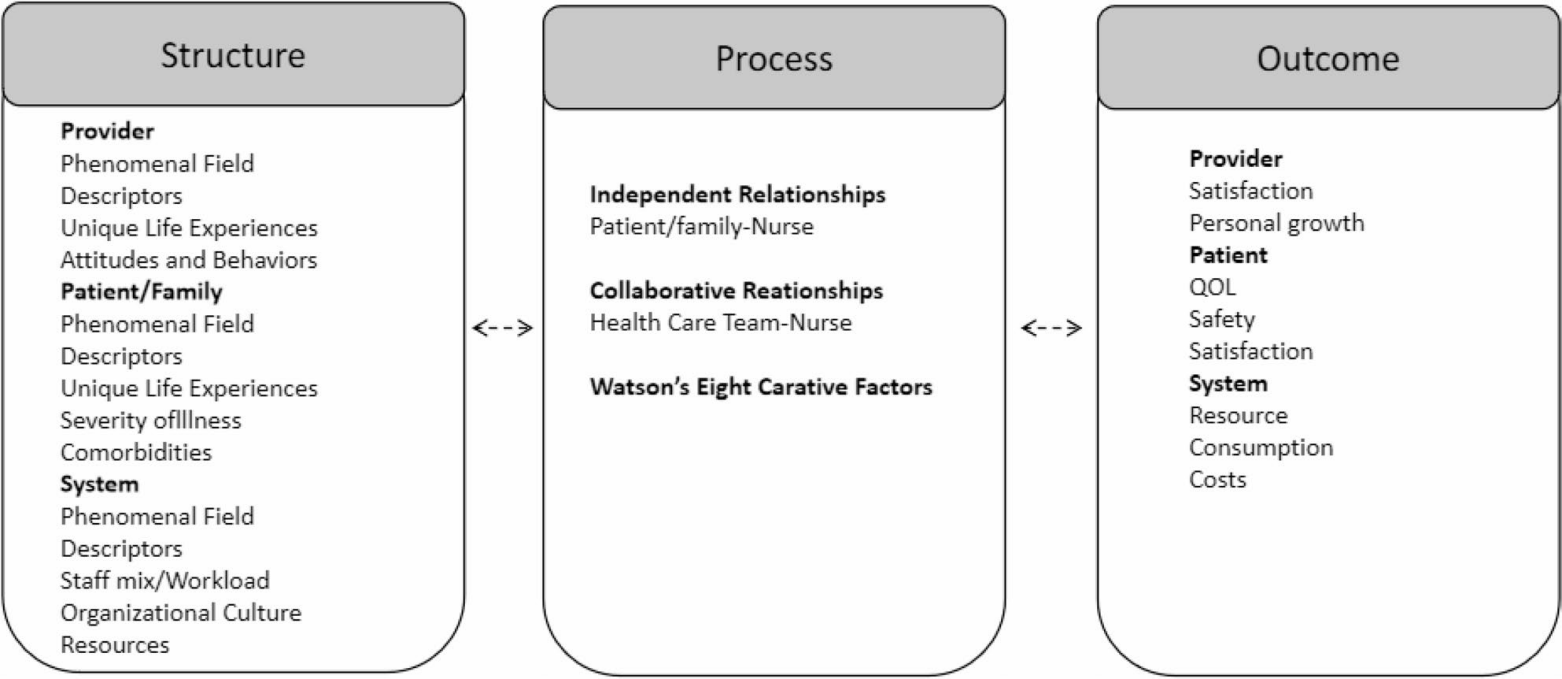
Conceptual illustration of the Quality Caring Model

The Quality Caring Model, proposed by Dr. Joanne Duffy in 2003, was developed on the basis of Watson’s theory of human caring and integrated with Donabedian’s three-dimensional quality framework of structure, process, and outcomes [31–33]. This model has been used as a theoretical foundation for the implementation of caring practices in clinical nursing and for the development of quality evaluation indicators related to caring nursing [34–37]. In the Quality Caring Model, structure refers to the principal parties involved in caring, including nursing staff who provide health services, patients and their families, and the healthcare system. Within the overall management of humanistic care quality, each of these parties possesses distinct characteristics, attributes, and lived experiences. Process refers to the behavioral interventions and professional practices implemented by healthcare providers during the course of care, with its core centered on the establishment and maintenance of caring relationships. The process dimension encompasses eight caring elements derived from Watson’s theory of human caring, namely the creation of a healing environment, collaborative problem-solving, recognition of human uniqueness, respect, encouragement, patient reassurance, fulfillment of basic human needs, and fulfillment of belonging needs. Outcomes refer to the results of healthcare services and constitute the feedback and control component of quality management. They include both intermediate and final outcomes. Intermediate outcomes denote changes in the cognition, emotions, and behaviors of nursing staff, patients, and their families, which may influence final care outcomes. Final outcomes refer to results with longer-term implications, such as quality of life and care satisfaction. Outcomes are dynamic and amenable to continuous improvement, and the achievement of favorable outcomes largely depends on the establishment and maintenance of caring relationships. The conceptual framework of the Quality Caring Model, illustrating the structure–process–outcome components, is presented in Fig. 2.

Maslow’s hierarchy of needs theory, proposed by the American scholar Abraham Maslow [38], emphasizes that human needs are organized in a hierarchical progression from lower to higher levels, including physiological needs, safety needs, needs for love and belonging, esteem needs, and self-actualization needs. Based on this theory, humanistic care should not only focus on meeting older adults’ basic living and safety needs but also place greater emphasis on higher-level needs such as emotional belonging, respect, and the realization of self-worth. This theoretical framework provides important support for the present study in comprehensively understanding the connotations and hierarchical structure of the quality of humanistic care in nursing homes.

Therefore, the Quality Caring Model (QCM) was adopted in this study as the overarching theoretical framework for the development of the evaluation indicator system of humanistic care quality in nursing homes, serving to define the structure–process–outcome dimensions of humanistic care quality and its caring connotations. On this basis, Maslow’s hierarchy of needs theory was incorporated to complement the analysis of the multidimensional needs of older adults in humanistic care services within nursing homes from a needs-based perspective, thereby providing theoretical support for the systematic development of the evaluation indicator system.

### Literature research

The research team conducted comprehensive literature searches across six databases—PubMed, EBSCO, Web of Science, China National Knowledge Infrastructure (CNKI), Wanfang, and VIP—using a combination of subject terms and free-text terms. References of the included studies and relevant review articles were also screened to identify additional literature. In parallel, policy documents were retrieved from official governmental websites, including the Chinese Government Network and the National Health Commission of China, using keywords such as “nursing homes,” “elderly care services,” and “humanistic care.” The search period for both literature and policy documents covered the time from database inception to October 2022. The inclusion criteria were as follows: (1) literature or policy documents related to geriatric nursing, nursing homes, humanistic care, evaluation indicators, or nursing quality management; (2) formally published literature in Chinese or English; and (3) policies that are currently in effect. The exclusion criteria were as follows: (1) notices, letters, conference abstracts, news reports, or laboratory-based basic research; and (2) studies for which the full text was unavailable. Through a comprehensive review of the literature and interpretation of relevant policy documents, the current status of research, implementation strategies, and existing challenges related to humanistic care in nursing homes were identified. Based on this background, indicators relevant to the evaluation of humanistic care quality in nursing homes were extracted and screened. These findings were then further integrated and refined at the theoretical level by drawing on the Quality Caring Model and Maslow’s hierarchy of needs theory, leading to the preliminary development of a draft indicator system comprising the core dimensions and sub-dimensions for evaluating the quality of humanistic care in nursing homes.

### Semi-structured field interview

A descriptive qualitative research design was adopted. Semi-structured interviews were conducted to explore the experiences and views of different groups regarding humanistic care in nursing homes, including older people’s lived experiences and perceptions of humanistic care, as well as care workers’, nurses’, and nursing managers’ suggestions concerning current humanistic care practices and quality evaluation indicators.

Based on a synthesis of the relevant literature, a brainstorming approach was employed, in which one expert in geriatric nursing and three members of the research team collaboratively discussed and developed the interview outline (Supplementary Material 1). Purposive sampling was then used to conduct on-site interviews between November 2022 and February 2023 among older residents, care workers, nurses, and nursing managers at a nursing home in Hangzhou. This nursing home was selected because it had relatively standardized management practices, stable staffing, and established experience in providing long-term care services, which enabled the research team to obtain information from multiple stakeholder groups involved in daily humanistic care practices. The interviews were not intended to generate representative conclusions for all nursing homes, but rather to supplement and refine the preliminary indicator framework developed from the literature review and policy analysis. To reduce potential site-specific bias, the interview findings and preliminary indicators were repeatedly discussed within the research team and subsequently examined and revised through Delphi expert consultation involving experts from different institutions and regions. Sample size determination followed the principle of data saturation in qualitative research [39], whereby data collection and analysis were conducted concurrently, and saturation was considered to be achieved when subsequent interviews yielded no new themes or information. An additional one to two participants were recruited to verify the stability of the saturation judgment. Ultimately, a total of 26 participants were included, comprising seven older residents, eight care workers, five nurses, and six nursing managers (Supplementary Material 1). Prior to the interviews, participants were fully informed about the study objectives, significance, and principles of data confidentiality, and were notified that the interviews would be audio-recorded. Written informed consent was obtained from all participants before participation. All interviews were conducted by the researcher in a quiet, private room using a one-to-one format, with no other individuals present besides the interviewer and the participant. During the interviews, participants were encouraged to express themselves freely, and all interview topics were explored in depth. The interviewer, who had received training in qualitative interviewing and field-note writing, made brief field notes during the interviews to record notable non-verbal cues, such as facial expressions, pauses, tone changes, and emotional responses. These notes were used as contextual information to support the interpretation of the interview transcripts and were not subjected to separate coding or independent thematic analysis. Each interview lasted approximately 30–50 min. Within 48 h after the completion of each interview, the audio recordings were transcribed verbatim into textual data by the researcher.

Interview data were analyzed using conventional content analysis, also referred to as inductive content analysis [40]. The researcher repeatedly read the interview transcripts to gain an overall understanding of the data, after which content reflecting key viewpoints and meaning units was identified and annotated, followed by open coding of the text. Codes with similar meanings or related content were then grouped into categories. The development of themes and sub-themes was an iterative process; as additional interview data were continuously incorporated, existing themes could be decomposed, revised, or supplemented with new themes. The draft evaluation indicator system was subsequently refined based on the findings of the qualitative interviews.

### Modification and determination of index system

The Delphi method, first developed in the 1940s, is an expert consultation technique [41]. It employs an anonymous, iterative communication process to solicit opinions from a selected panel of experts over multiple rounds. Through repeated summarization, synthesis, and revision of expert feedback, the opinions of the expert panel gradually converge, ultimately leading to a relatively consistent conclusion or judgment. This study further refined the indicator items through Delphi expert consultation. Experts were purposively selected from the fields of geriatric nursing, nursing management, and humanistic nursing. The inclusion criteria were as follows: (1) having at least 10 years of experience in geriatric nursing, nursing management, or humanistic nursing, or being engaged full-time in the management of nursing homes; (2) holding an intermediate or higher professional title; (3) possessing a bachelor’s degree or above; and (4) voluntary participation in this study. The exclusion criterion was failure to return the questionnaire within the specified time frame. The number of experts involved in a Delphi consultation is typically influenced by factors such as the scope of the study, and it is generally considered that the participation of 15–50 experts represents an appropriate range [42]. Taking into account the scale, feasibility, and accessibility of the present study, and in accordance with the expert inclusion criteria, a total of 34 experts from relevant fields were invited to participate.

After the target expert panel was identified, the research team developed the expert consultation questionnaire. Prior to questionnaire distribution, experts were contacted via email, telephone, or WeChat to introduce the study background, objectives, methods, and content. Upon obtaining their consent, the questionnaire was distributed via email or WeChat. To ensure the timeliness and effectiveness of data collection, invited experts were asked to complete the questionnaire within two weeks of receipt. The questionnaire consisted of four sections: an introduction to the questionnaire, a survey of expert characteristics, an evaluation questionnaire for the preliminary indicator system, and a self-assessment form in which experts rated their familiarity with the consultation content (Cs) and the basis for their judgments (Ca) (Supplementary Material 2). In the evaluation of the preliminary indicator system, experts were asked to rate the importance of each indicator listed in the consultation questionnaire using a 5-point Likert scale (1 = unimportant, 2 = slightly unimportant, 3 = moderately important, 4 = relatively important, and 5 = very important), and to provide relevant suggestions. Based on the results of the expert consultation, the research team reviewed, synthesized, and analyzed the indicators, retaining those with a mean importance score ≥ 4.0, a coefficient of variation (CV) < 25%, and a percentage of full scores > 20% [43]. In combination with expert feedback, the indicators were further screened and refined, and the second-round expert consultation questionnaire was subsequently developed. In the second-round questionnaire, experts were required to provide basic information, rate the importance of the evaluation indicators, and complete self-assessments of their familiarity with the indicators (Cs) and the basis for their judgments (Ca).

### Index weight determination

The Analytic Hierarchy Process (AHP) is a systematic method for multi-criteria decision-making [44]. It decomposes complex decision problems into an ordered hierarchical structure and quantifies the relative importance of each factor through mathematical calculations, thereby providing a basis for decision-making. In this study, yaahp version 10.3 software was used to construct the analytical structure of the evaluation indicator system for the quality of humanistic care in nursing homes based on the Analytic Hierarchy Process.

### Establish the hierarchical structure model

When constructing the hierarchical model, the evaluation indicator system was divided into three levels: the target layer, the criterion layer, and the indicator layer. The target layer represents the highest level of the hierarchy and reflects the overall decision-making objective or ideal outcome of the problem, namely the humanistic care quality evaluation indicator system in nursing homes. The criterion layer represents the intermediate steps required to achieve this objective and may include multiple sub-levels. In this study, the criterion layer comprised two levels, including the primary-level indicators and the secondary-level indicators. The indicator layer specifies the concrete measures for achieving the research objective and corresponds to the tertiary-level indicators in the evaluation system of this study.

The decision to structure the evaluation system into primary-, secondary-, and tertiary-level indicators was based on the multidimensional and hierarchical nature of humanistic care quality in nursing homes. Primary-level indicators were used to represent the core dimensions of humanistic care quality, secondary-level indicators further specified the main aspects within each core dimension, and tertiary-level indicators translated these domains into concrete, observable, and relatively measurable items linked to specific care practices and management elements. This hierarchical structure helped operationalize the abstract concept of humanistic care into specific evaluation content and provided a clear basis for subsequent weight determination using the Analytic Hierarchy Process.

### Building the judgment matrix

When constructing judgment matrices at each hierarchical level, the traditional Analytic Hierarchy Process typically requires experts to perform pairwise comparisons of indicators within the same level using Saaty’s 1–9 scale. However, when the number of indicators is large, this approach can substantially increase the cognitive burden on experts and may adversely affect the consistency of their judgments. Therefore, drawing on approaches reported in previous studies [45–48], this study calculated the mean differences in importance scores among indicators within the same hierarchical level based on the results of the final round of Delphi expert consultation, and determined the corresponding Saaty’s 1–9 scale values accordingly to construct the pairwise comparison judgment matrices. The correspondence between the mean differences and the Saaty scale values is presented in Table 1.

**Table 1:** Correspondence between mean differences in importance scores and Saaty’s 1–9 scale

Mean difference in importance scores (Aij − Aji)	Verbal judgment	Numerical Value (Saaty scale)
*A*_*i*j_ - *A*_*ji*_ = 0	Aij and Aji are of equal importance	1
0.25 < *A*_*i*j_ - *A*_*ji*_ ≤ 0.50	Aij is slightly more important than Aji	3
0.75 < *A*_*i*j_ - *A*_*ji*_ ≤ 1.00	Aij is much more important than Aji	5
1.25 < *A*_*i*j_ - *A*_*ji*_ ≤ 1.50	Aij is very strongly more important than Aji	7
1.75 < *A*_*i*j_ - *A*_*ji*_	Aij is extremely more important than Aji	9

Aij and Aji denote the mean importance ratings assigned by the experts to two different indicators at the same hierarchical level. When the calculated difference falls between two adjacent Saaty scale categories, intermediate values of 2, 4, 6, or 8 are assigned accordingly

### Weight coefficient calculation and consistency test

Yaahp version 10.3 software was used to calculate the weights of indicators at each hierarchical level. Based on the constructed hierarchical model, the local weights of indicators within their respective levels were first computed. Subsequently, according to the weight synthesis rules of the Analytic Hierarchy Process, the composite weights of each indicator relative to the overall objective were further calculated. Specifically, the composite weight of each secondary-level indicator was obtained by multiplying its local weight by the weight of the corresponding higher-level indicator, according to the formula: *W*_*ij*_ = *w*_*ij*_*×W*_*i* ,_ where *W*_*ij*_ represents the composite weight of the *j* secondary-level indicator under the *i* higher-level indicator, *w*_*ij*_ denotes the local weight of the secondary-level indicator within its respective level, and *W*_*i*_ is the weight of the corresponding higher-level indicator. For multi-level indicator systems, weight synthesis was performed sequentially in a top-down manner, ultimately yielding the composite weights of the lowest-level indicators relative to the overall objective; for primary-level indicators, their local weights were equal to their composite weights. In addition, a consistency test was performed for each judgment matrix by calculating the maximum eigenvalue (λmax) and deriving the consistency ratio (CR). A judgment matrix was considered to have acceptable consistency when CR < 0.10, indicating that the weighting scheme was reasonable.

### Data statistics and analysis

Statistical analysis and description of the data obtained from the expert consultation were conducted using IBM SPSS Statistics 25.0 and Excel 2021. The general information of the experts was analyzed descriptively; the experts’ enthusiasm was represented by the questionnaire response rate; the experts’ authority was represented by the authority coefficient (Cr), which was calculated as the arithmetic mean of familiarity (Cs) and judgment basis (Ca), i.e., Cr = (Ca + Cs) / 2. The Cr value ranges from 0 to 1, with higher Cr values indicating greater reliability of the consultation results. A Cr value of ≥ 0.7 is generally considered acceptable [49]. The concentration degree of expert opinion was evaluated according to the mean of the expert importance value of each item, the coefficient of variation (CV), and the full score ratio. The degree of coordination of expert opinions was mainly represented by Kendall’s coefficient of concordance (Kendall’s W). Kendall’s W ranges from 0 to 1.0, with higher values indicating better coordination; *P* < 0.05 was considered statistically significant. A structural hierarchy model was established using yaahp 10.3 software, judgment matrices were constructed, the weights of each indicator were determined, and consistency tests were performed.

## Results

### Draft development of the evaluation indicator system

Based on the Quality Caring Model and Maslow’s Hierarchy of Needs, and informed by findings from the literature review and semi-structured interviews, the research team repeatedly discussed and screened the indicators, removing those with overlapping meanings or unclear definitions. As a result, a preliminary evaluation indicator system for humanistic care quality in nursing homes was established, comprising three primary-level indicators, 14 secondary-level indicators, and 70 tertiary-level indicators.

### Basic information about the experts

A total of 32 experts completed two rounds of consultation. The experts were aged 38–59 years (mean = 48.59, SD = 6.72) and had 14–41 years of professional experience (mean = 28.16, SD = 8.19). Regarding educational background, 22 experts (68.75%) held a bachelor’s degree, 6 (18.75%) held a master’s degree, and 4 (12.50%) held a doctoral degree. In terms of professional titles, 6 experts (18.75%) held intermediate titles, 11 (34.38%) held associate senior titles, and 15 (46.88%) held senior titles. With respect to research areas, 9 experts (28.13%) were engaged in geriatric nursing research, 10 (31.25%) in nursing management research, 5 (15.63%) in nursing humanistic care research, and 8 (25.00%) in older care service management research. Detailed information on the experts is presented in Table 2.

**Table 2:** Demographic information of experts (*n* = 32)

Items	*N* (%)
Gender	
Male	2 (6.25)
Female	30 (93.75)
Age (years)	
30 ~ 39	2 (6.25)
40 ~ 49	15 (46.88)
50 ~ 59	15 (46.88)
Education level	
Bachelor degree or below	22 (68.75)
Master’s degree	6 (18.75)
PhD degree	4 (12.50)
Professional title	
Middle	6 (18.75)
Associate senior	11 (34.38)
Senior	15 (46.88)
Work experience (years)	
10～19	6 (18.75)
20～29	10 (31.25)
≥ 30	16 (50.00)
Research field	
Geriatric nursing	9 (28.13)
Nursing management	10 (31.25)
Humanistic nursing	5 (15.63)
Elderly care services and management	8 (25.00)

Professional titles are based on China’s nationally standardized academic and healthcare ranking system. Middle refers to an intermediate-level position (e.g., Lecturer or Attending Physician/Nurse), associate senior refers to an associate senior position (e.g., Associate Professor or Associate Chief Physician/Nurse), and senior title refers to the highest professional rank (e.g., Full Professor or Chief Physician/Nurse)

### Enthusiasm of experts and the degree of authority of experts

A total of two rounds of expert consultation were conducted in this study. In the first round, 34 experts were invited, of whom 2 did not return the questionnaire within the specified time, resulting in 32 valid responses and a valid response rate of 94.12%. The second round of consultation was conducted with the 32 experts who had completed the first round, and all experts returned the questionnaires within the specified time, yielding a valid response rate of 100.00%. The authority coefficient (Cr) was 0.850 for the first round and 0.859 for the second round. The results of the experts’ judgment basis and their familiarity with the consultation content are shown in Table 3.

**Table 3:** Expert authority coefficient

Rounds	Ca	Cs	Cr
Round 1	0.925	0.775	0.850
Round 2	0.906	0.813	0.859

### Coordination degree of expert opinions

As shown in Table 4, the Kendall’s W values for the indicators in the two rounds of Delphi consultation were 0.167 and 0.269, respectively. A comparison of the Kendall’s W values between the two rounds of consultation showed that the differences were statistically significant (χ² = 458.519, 447.035, *P* < 0.001).

**Table 4:** Expert coordination coefficient

Round	Hierarchical level	Items (*n*)	Kendall’s W	χ^2^	*P*
Round 1	Primary-level	3	0.102	6.500	0.039
	Secondary-level	14	0.113	46.886	< 0.001
	Tertiary-level	70	0.169	373.011	< 0.001
	Total	87	0.167	458.519	< 0.001
Round 2	Primary-level	3	0.094	6.000	0.050
	Secondary-level	10	0.390	124.759	< 0.001
	Tertiary-level	40	0.230	280.228	< 0.001
	Total	53	0.269	447.035	< 0.001

### The formation process of the evaluation index system

In the first round of expert consultation, no modifications were proposed for the primary-level indicators, and therefore all primary-level indicators were retained. With respect to the secondary-level indicators, revisions were made by the research team based on expert feedback to enhance the logical consistency and standardization of the indicator system. Specifically, four experts pointed out that the indicator “Older people and their family members” under structural quality overlapped with the same indicator under outcome quality and recommended its removal. Additionally, two experts pointed out that the credibility of self-assessment by nursing homes was relatively low and recommended the removal of the indicator “Nursing homes” under outcome quality. After discussion by the research team, this suggestion was adopted. Furthermore, three experts suggested that the indicators “Meeting daily living care needs,” “Meeting physical health care needs,” and “Meeting spiritual and cultural care needs” all correspond to the fulfillment of basic needs within the Quality Caring Model and recommended consolidating these indicators into a single indicator, “Meeting basic daily living care needs,” which was also adopted. In response to expert recommendations, the research team revised the descriptions of six indicators. For the tertiary-level indicators, the research team made adjustments based on expert feedback and group discussions: 20 items were removed, 26 items were consolidated into 9, 6 new items were added, and 23 item descriptions were revised, as detailed in Supplementary Materials 3. Following these revisions, a final evaluation indicator system for humanistic care quality in nursing homes was established, consisting of 3 primary-level indicators, 11 secondary-level indicators, and 40 tertiary-level indicators, which were included in the second round of expert consultation.

**Fig. 3:**
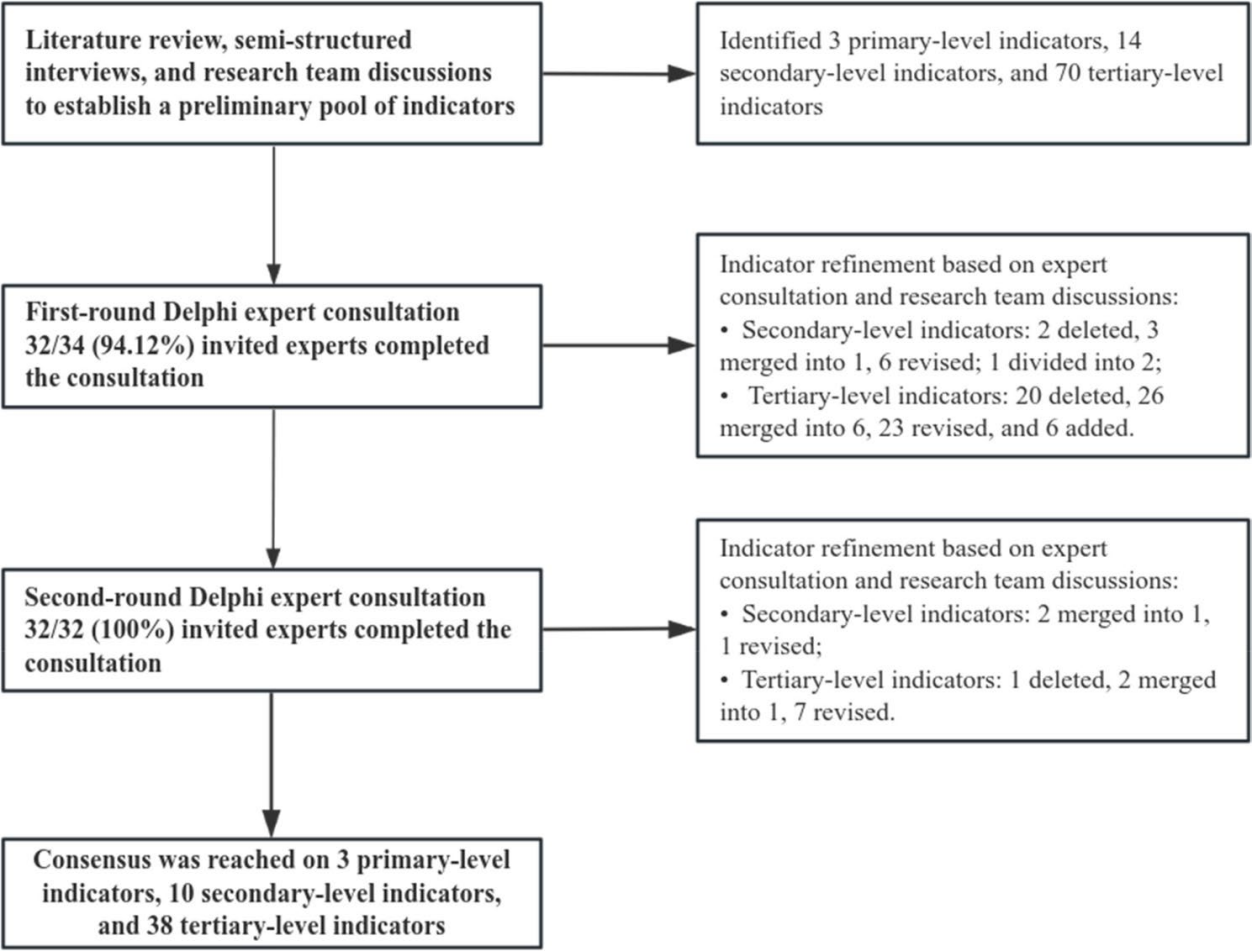
The process of developing the evaluation indicator system for humanistic care quality in nursing homes

In the second round of expert consultation, the experts’ opinions on the indicators at all levels were generally consistent, and no new modifications were proposed for the primary-level indicators, which were therefore retained. For the secondary-level indicators, one expert suggested changing “Appreciation of individual uniqueness” to “Appreciation of older people’s self-worth” to reflect the essence of humanistic care more accurately. Additionally, considering the similarity in the evaluation subjects and content between “Evaluation by older people” and “Evaluation by family members,” two experts recommended merging the two indicators into “Evaluation by older people and their family members,” which was adopted after discussion by the research team. At the tertiary level, the research team further refined and standardized the indicators based on expert feedback, including the removal of one item and the revision of seven item descriptions to enhance clarity and operability. Through repeated feedback and revisions over two rounds of Delphi expert consultation, the structure and content of the indicator system were further improved, resulting in the final evaluation indicator system for humanistic care quality in nursing homes, which consists of 3 primary-level indicators, 10 secondary-level indicators, and 38 tertiary-level indicators. The flowchart is shown in Fig. 3.

In this study, the results of the expert consultation were integrated with the Analytic Hierarchy Process to calculate the weights of the indicators and to conduct consistency tests. The consistency ratios of indicators at all hierarchical levels were less than 0.1000, as shown in Table 5 and Supplementary Material 4.

**Table 5:** Humanistic care quality indicator system for older people in nursing homes and weight values for each indicator

Indicators	Initial weight	Composite weight
1 Structure	0.311	0.311
1.1 Humanistic care system	0.558	0.174
1.1.1 Establishment of humanistic caring quality management groups at all levels	0.164	0.028
1.1.2 Develop humanistic caring workflows, practice guidelines, and inspection standards	0.448	0.078
1.1.3 Rationally allocate the number of elderly care nursing staff based on older people’s functional assessment levels and care needs	0.283	0.049
1.1.4 Establish an organizational and management system for volunteer services	0.106	0.018
1.2 Nursing workforce development	0.320	0.099
1.2.1 Establish appropriate values for elderly care services and a humanistic caring philosophy	0.048	0.005
1.2.2 Provide training in humanistic caring knowledge and skills	0.315	0.031
1.2.3 Conduct assessment and continuous improvement of humanistic caring knowledge and skills	0.198	0.020
1.2.4 Establish incentive mechanisms for humanistic caring practices and safeguard nursing staff welfare and benefits	0.094	0.009
1.2.5 Implement activities to support and care for nursing staff	0.250	0.025
1.2.6 Foster a harmonious and mutually supportive work environment	0.094	0.009
1.3 Caring environment	0.122	0.038
1.3.1 Create a warm and comfortable living environment	0.330	0.013
1.3.2 Ensure environmental safety and privacy protections	0.200	0.008
1.3.3 Appropriate configuration of various functional spaces	0.330	0.013
1.3.4 Provide nostalgic emotional value	0.140	0.005
2 Process	0.493	0.493
2.1 Meeting basic daily living care needs	0.296	0.146
2.1.1 Provide basic daily living care	0.231	0.034
2.1.2 Implement safety assurance measures	0.490	0.072
2.1.3 Provide health care services	0.163	0.024
2.1.4 Provide leisure and recreational activities	0.116	0.017
2.2 Communication and comfort	0.296	0.146
2.2.1 Emphasize humanistic caring in communication	0.667	0.097
2.2.2 Assess older people’s psychological status and provide psychological comfort	0.333	0.049
2.3 Meeting care needs for love and belonging	0.151	0.074
2.3.1 Build harmonious interpersonal relationships between nursing staff and older people	0.122	0.009
2.3.2 Attend to and coordinate interpersonal relationships among older people	0.312	0.023
2.3.3 Assisting older people in maintaining contact with their families	0.122	0.009
2.3.4 Carry out volunteer services to support older people	0.444	0.033
2.4 Respect and encouragement	0.200	0.099
2.4.1 Respect older people’s cultural customs and religious beliefs	0.182	0.018
2.4.2 Respect and protect older people’s bodily privacy and personal information privacy	0.332	0.033
2.4.3 Respect older people’s personal dignity and avoid infantilizing cognitively intact older people	0.064	0.006
2.4.4 Respect for older people’s right to be informed and encouragement of shared care planning	0.102	0.010
2.4.5 Respect older people’s right to supervision and encourage shared participation in institutional management	0.239	0.024
2.4.6 Encourage self-care among older people, emphasizing attention to personal health and the adoption of healthy behaviors	0.081	0.008
2.5 Appreciation of older people’s self-worth	0.057	0.028
2.5.1 Pay attention to older people’s life backgrounds and their views on life, the world, and values	0.250	0.007
2.5.2 Appreciate and affirm older people’s past achievements and current performance	0.250	0.007
2.5.3 Identify and cultivate older people’s interests and strengths	0.500	0.014
3 Outcome	0.196	0.196
3.1 Evaluation by older people and their family members	0.750	0.147
3.1.1 Older people’s satisfaction with humanistic caring	0.625	0.092
3.1.2 Evaluation of older people’s quality of life	0.239	0.035
3.1.3 Family members’ satisfaction with humanistic care	0.137	0.020
3.2 Evaluation by nursing staff	0.250	0.049
3.2.1 Nursing staff’s self-evaluation of humanistic caring quality	0.800	0.039
3.2.2 Work well-being index of nursing staff	0.200	0.010

## Discussion

Humanistic care plays a crucial role in meeting the comprehensive care needs of older adults and serves as an important indicator for achieving person-centered services and promoting the development of a family-like care environment. As an essential component of the aged care service system, the improvement of care quality in nursing homes is closely linked to the implementation of humanistic care philosophy. Effectively improving the standard of humanistic care and ensuring its quality requires a scientific and standardized evaluation system. Therefore, developing a quality evaluation indicator system for humanistic care in nursing homes is not only a proactive response to the needs of the older population but also an essential requirement for nursing homes to achieve high-quality development.

### Scientific rigor and reliability assessment of the humanistic care quality evaluation indicator system in nursing homes

Based on the Quality Caring Model, this study constructed an evaluation framework from three dimensions: structure, process, and outcomes. The preliminary indicators were derived from a previously developed indicator pool for evaluating the quality of humanistic care in nursing homes. This item pool was established through a systematic review of relevant domestic and international evaluation frameworks, measurement tools, and policy documents, in combination with findings from semi-structured interviews, and was refined through group discussions to ensure the comprehensiveness and scientific rigor of the indicators. Subsequently, the research team developed an expert consultation questionnaire and invited experts to rate the importance of the indicators and provide feedback. Following group discussions, indicators were retained, removed, or modified, resulting in a final evaluation indicator system comprising 3 primary-level indicators, 10 secondary-level indicators, and 38 tertiary-level indicators.

The scientific rigor of the indicator system was supported by the multidisciplinary composition and professional authority of the Delphi expert panel. The experts were drawn from fields closely related to humanistic care quality in nursing homes, including geriatric nursing, humanistic nursing, nursing management, and elder care services. This composition helped ensure that the indicators were assessed from both theoretical and practical perspectives, thereby strengthening the content validity and practical relevance of the evaluation indicator system.

In the two rounds of expert consultation, the questionnaire response rates were 94.12% and 100.00%, respectively, indicating a high level of expert engagement. In this study, the authority coefficients (Cr) for the two rounds of consultation were 0.850 and 0.859, both greater than 0.80, indicating a high level of expert authority and strong reliability of the Delphi consultation results. In the two rounds of expert consultation, Kendall’s W coefficients were 0.167 and 0.269, respectively. Although these values did not approach 1, within the context of a Delphi study they reflect reasonable disagreement among experts at the initial stage, followed by a trend toward convergence of opinions. Considering that more than 20 experts participated in this study and the number of evaluation indicators was relatively large (87 indicators in the first round and 53 indicators in the second round), a chi-square test was conducted to assess statistical significance [50, 51]. The results were statistically significant (first round: χ² = 458.519, *P* < 0.001; second round: χ² = 447.035, *P* < 0.001), indicating that expert opinions were relatively consistent and showed good coordination, thus demonstrating a high level of reliability in the Delphi consultation results. In addition, the weights of the indicators for humanistic care quality in nursing homes were determined using the Analytic Hierarchy Process. Each judgment matrix constructed was tested for consistency, with consistency ratios being less than 0.1, indicating good consistency in the judgment matrices and scientifically sound weight assignments.

### Content analysis of the evaluation indicator system

This study constructed an evaluation indicator system for the quality of humanistic care in nursing homes based on three dimensions: structural quality, process quality, and outcome quality. The system also integrates the eight caring factors proposed by the humanistic care theory and Maslow’s Hierarchy of Needs, ensuring the systematic and comprehensive nature of the evaluation content. Regarding weight assignment, the weights for structural quality, process quality, and outcome quality were 0.3108, 0.4934, and 0.1958, respectively. Process quality (0.4934) has the highest weight among the primary-level indicators, indicating that the focus of the evaluation of humanistic care quality in nursing homes is primarily on the implementation of humanistic care in daily caregiving activities. As the central component of quality management and control, process quality reflects how care staff implement person-centered principles through specific caregiving behaviors and service interactions, respond to the multidimensional needs of older people, and demonstrate concern for their overall life and health. At the same time, process quality is also a key factor influencing the caregiving experience of older people. Furthermore, the evaluation of structural quality and outcome quality is equally important. Based on the Quality Caring Model, which incorporates Donabedian’s Structure-Process-Outcome framework, there is a positive relationship between structure, process, and outcomes. That is, good structure provides the foundation for high-quality care processes, while high-quality care processes contribute to achieving ideal care outcomes [31, 33]. By monitoring and analyzing the quality of outcomes, it is possible to provide feedback to guide structural configuration and process optimization, further promoting the improvement and enhancement of humanistic care quality in nursing homes.

Structural quality ensures the feasibility of implementing humanistic care services and serves as a critical foundation for the effective operation of the humanistic care system. In this study, the evaluation of structural quality includes three secondary-level indicators: “Humanistic care system” (0.5584), Nursing workforce development (0.3196), and Caring environment (0.1220), with the “Humanistic care system” having the highest weight (0.5584). Among the tertiary-level indicators under this domain, “Develop humanistic caring workflows, practice guidelines, and inspection standards” received the highest weight, a finding that is consistent with China’s policy orientation toward promoting high-quality nursing services [52]. International studies have also emphasized that person-centered nursing home care requires organizational and system-level support rather than relying only on individual staff behaviors [53]. Evidence further suggests that structured quality improvement frameworks can contribute to tangible improvements in care quality: a Dutch government-funded programme demonstrated measurable gains in nursing homes with urgent quality issues [54], and a systematic review confirmed that quality improvement strategies have been broadly adopted to support monitoring and guide improvement across care home settings, though implementation effects vary [55]. Establishing and refining an organizational framework for humanistic care quality management, clarifying humanistic care workflows, and formulating detailed practice guidelines and inspection standards can therefore provide clear guidance for the systematic and sustained implementation of humanistic care services in nursing homes. Another tertiary-level indicator with a relatively high weight was “rationally allocate the number of elderly care nursing staff based on older people’s functional assessment levels and care needs”. The *Specifications for Post Setting and Staffing Allocation in Nursing Homes* issued by the Ministry of Civil Affairs of the People’s Republic of China [56] clearly states that the number of nursing staff should be reasonably allocated based on older people’s functional assessment levels and care needs. Previous studies have consistently demonstrated that the rationality of staff allocation is a key factor influencing the quality of care in nursing homes. For instance, Gorges and Konetzka [57] found that nursing homes with lower staffing levels experienced significantly more COVID-19 cases and outbreaks, highlighting how inadequate staffing directly compromises resident safety. Similarly, Glette et al. [58] reported that nursing home leaders and nurses perceived insufficient staffing and competence levels as major contributors to unplanned hospital readmissions, underscoring the broader impact of workforce allocation on care continuity and outcomes. Song and Song [59] further demonstrated that staff mix, especially the proportion of registered nurses, was positively associated with nursing home quality across nursing homes in Korea. These findings provide empirical support for considering staffing allocation, staff competence, and staff mix when evaluating the structural quality of humanistic care in nursing homes. The findings of this study are also consistent with those reported by Jadidi et al. in the development of the Nursing Home Accreditation Scale [60], which incorporates human resource allocation as a structural evaluation dimension, thereby indirectly supporting the critical role of staffing allocation in the assessment of care quality in aged care services.

Process quality is an important dimension for assessing the quality of humanistic care in nursing homes and serves as a key link between structural conditions and outcome indicators. Among the secondary-level indicators in process quality, “Meeting basic daily living care needs” and “Communication and comfort” both have the highest and identical combined weights (0.296), indicating that nursing homes should not only focus on the basic physiological and safety needs of older people but also give equal attention to their emotional and psychological needs. The indicator “Meeting basic daily living care needs” is consistent with Maslow’s hierarchy of needs theory, indicating that nursing homes should first ensure that the basic daily care needs of older people are met, on the basis of which greater attention can then be directed toward addressing their psychological and social needs; therefore, this indicator occupies an important position in the evaluation of process quality. The tertiary-level indicator with the highest weight in this dimension is “Implement safety assurance measures”. In recent years, safety issues in nursing home care have become a focal point in the aged care industry, with incidents such as falls, pressure ulcers, choking, and aspiration frequently occurring among older people. Safety management is fundamental to ensuring the quality of life for older people and should receive high priority in nursing homes. At the same time, due to changes in living environments, declines in self-care abilities, and shifts in social roles, older people often experience varying degrees of loneliness, depressive emotions, and a lack of emotional support [61]. In this context, “Communication and comfort” play an irreplaceable role in meeting the care and caregiving needs of older people. Nursing homes should emphasize humanistic care in communication processes, skillfully apply both verbal and non-verbal communication techniques, and regularly assess the psychological state of older people, providing necessary psychological support to practice a person-centered care philosophy.

Outcome quality primarily reflects the effects achieved following the implementation of humanistic care in nursing homes. Among the outcome quality indicators, “Evaluation by older people and their family members” has the highest weight (0.7500). The fundamental aim of humanistic care quality management is to maximize older people’s care experiences and satisfaction through standardized management and professional care practices, thereby promoting continuous improvement. From a quality management perspective, the ultimate evaluation of service quality should be centered on the service recipients; older people’s satisfaction not only directly reflects the overall effectiveness of humanistic care services but also effectively reveals issues and deficiencies in care management. By systematically collecting evaluation results from older people, their families, and caregivers, nursing home managers can more accurately identify weak areas, analyze the relationship between these areas and structural configuration and care processes, and develop targeted improvement measures.

### Implications

The evaluation indicator system and its weight distribution developed in this study may provide a reference for the systematic management and continuous improvement of humanistic care quality in nursing homes. As a quality evaluation tool at the institutional level, it can be used for exploratory assessments of humanistic care quality management. It may help managers systematically identify key domains, recognize potential weak areas in current care practices, and prioritize quality improvement efforts. In addition, the system can provide a basis for comparative analyses of humanistic care practices across different types of nursing homes and for subsequent empirical studies. It may also serve as a reference for the Ministry of Civil Affairs and other relevant administrative bodies in conducting research on humanistic care quality and formulating related policies.

### Limitations and future directions

This study has certain limitations. First, the 26 participants in the interviews were all from a single nursing home in Hangzhou. Compared to many other regions in China, the elderly care service system in Hangzhou is relatively well-developed, and the management practices in nursing homes are generally more standardized, with better staffing conditions. This institutional and resource background may have influenced the participants’ perceptions and experiences of humanistic care, potentially shaping the themes and preliminary indicators formed during the qualitative interviews. Therefore, the findings of this study may not fully represent the actual state of humanistic care quality assessment in nursing homes across China. Second, the experts involved in the Delphi consultation were mainly from regions in China, including Zhejiang, Sichuan, Shanghai, and Xinjiang. This regional concentration may, to some extent, limit the generalizability of the indicator system across areas with different levels of elderly care service development. In addition, as all consulted experts were from China, the applicability of this indicator system still needs to be further validated in other countries and cultural contexts; nevertheless, it may provide useful references for the evaluation of humanistic care quality in nursing homes internationally. Finally, due to time and resource constraints, the indicator system developed in this study remains at the theoretical construction stage, and empirical validation has not yet been conducted.

Given the aforementioned limitations, the research team plans to develop a survey tool based on the existing indicator system in the next phase of the study to systematically collect empirical data on humanistic care quality in nursing homes. After the development of the survey tool, multi-center pilot testing and formal surveys will be conducted in nursing homes of different regions and types. Through empirical data, the reliability, validity, and structural stability of the questionnaire will be systematically tested, and its applicability and generalizability across different institutional settings will be assessed. Based on this, the indicator descriptions, dimensional structure, and scoring criteria will be revised and improved in conjunction with actual measurement results and user feedback, making the evaluation tool more closely aligned with the real management scenarios of humanistic care in nursing homes. Further research will explore the application of this evaluation tool in the daily quality management and assessment practices of nursing homes. Through cross-sectional comparisons and longitudinal tracking analysis, the sensitivity and guiding value of the evaluation system for quality improvement will be validated. Additionally, the system will be continuously optimized through multiple rounds of empirical testing and practical application, enhancing its relevance, feasibility, and sustainability.

## Conclusions

Guided by the Quality Caring Model and Maslow’s hierarchy of needs as the theoretical framework, this study employed a literature review, semi-structured interviews, the Delphi method, and the analytic hierarchy process to systematically and comprehensively construct an evaluation indicator system for the quality of humanistic care in nursing homes. The indicator system comprises three first-level indicators, ten second-level indicators, and thirty-eight third-level indicators. The findings provide a foundational framework and indicator basis for the subsequent development of specific measurement instruments and the conduct of empirical studies, thereby contributing to the standardized evaluation of humanistic care quality. Moreover, this study offers theoretical references and methodological insights for different countries and regions in developing evaluation systems for humanistic care quality that are aligned with local cultural contexts and long-term care service settings, thus supporting the achievement of the goals of active aging and healthy aging.

## Supplementary Information

Supplementary Material 1.

Supplementary Material 2.

Supplementary Material 3.

Supplementary Material 4.

Supplementary Material 5.

## Data Availability

Data sharing is available upon reasonable request during the present study. Readers can contact Qin Shen (zaixiaoyanjiusheng@163.com) to submit raw data.
