# Supplementary material for: Construction of an evaluation indicator system for humanistic care quality in nursing homes
Source: BMC Geriatr. 2026 May 8;26:899. doi: 10.1186/s12877-026-07623-3 (PMC13330427; doi:10.1186/s12877-026-07623-3)
Supplement: Supplementary file 1 — Supplementary Material 1. [file 12877_2026_7623_MOESM1_ESM.docx]

**1. Inclusion and exclusion criteria for interview participants:**

Inclusion criteria for older people were as follows: (1) age ≥ 60 years; (2) residence in a nursing home for at least 6 months; and (3) voluntary participation in the study. Exclusion criteria were: (1) a diagnosis of dementia or other mental disorders; (2) inability to communicate normally; and (3) withdrawal from the study for any reason during the study period.

Inclusion criteria for care workers were as follows: (1) age ≥ 18 years; (2) holding a valid Geriatric Care Worker Certificate; (3) being directly responsible for elderly care with at least 2 years of experience; (4) adequate verbal communication skills and willingness to share perspectives; and (5) voluntary participation in the study. Exclusion criteria were: (1) temporary or relief staff; and (2) withdrawal from the study for any reason during the study period.

Inclusion criteria for nurses were as follows: (1) being licensed and actively practicing; (2) having at least 2 years of clinical experience; and (3) voluntary participation in the study. Exclusion criteria were: (1) temporary or relief nurses; and (2) withdrawal from the study for any reason during the study period.

Inclusion criteria for nursing managers were as follows: (1) currently managing nursing services; (2) having at least 2 years of managerial experience; and (3) voluntary participation in the study. Exclusion criteria were: (1) externally appointed honorary positions; (2) part-time managers; and (3) withdrawal from the study for any reason during the study period.

**2. Interview outline**

**The interview outline for older people includes the following questions:**

1. What kind of institutional environment makes you feel comfortable and gives you a sense of home?

(2) What language, attitude, and behaviors of the nursing staff make you feel cared and perceive them as trustworthy and reliable?

(3) Since moving into this nursing home, have you noticed any changes in your health, emotions, or daily life? If so, what factors do you think contribute to these changes? Could you provide an example?

(4) What additional care and support would you like to receive in nursing homes?

(5) In your opinion, what aspects of the current humanistic care services in nursing homes need improvement?

**The interview outline for care workers includes the following questions:**

(1) How do you carry out humanistic care in the process of caring for the elderly? How do you feel during this process?

(2) How do you think you should improve your caring ability?

(3) What challenges or difficulties do you encounter when implementing humanistic care for the elderly? How do you address these challenges, or what kind of support do you hope to receive?

(4) What suggestions do you have for improving humanistic care services in nursing homes?

**The interview outline for nurses includes the following questions:**

(1) What kind of organizational culture, human resources, and environmental facilities do you believe enhance humanistic care quality in nursing homes?

(2) How do you carry out humanistic care in the process of caring for the elderly? How do you feel during this process?

(3) In your opinion, what measures are important in the process of humanistic care?

(4) What aspects should be considered when evaluating the quality of humanistic care in nursing homes, in your opinion?

(5) Do you have any suggestions for the nursing homes to better carry out humanistic care?

**The interview Outline for nursing managers:**

(1) What kind of organizational culture, human resources, and environmental facilities do you think contribute to the improvement of humanistic care quality in nursing homes?

(2) What do you think is humanistic care? Has your nursing homes already implemented humanistic care?

(3) As a nursing manager, what challenges do you encounter in implementing humanistic care in nursing homes? How have these challenges been addressed?

(4) What methods does your nursing home use to evaluate the quality and effectiveness of humanistic care?

(5) In your opinion, what aspects should be included in the evaluation of the quality and effectiveness of humanistic care in elderly care institutions?

**3. Sociodemographic characteristics of interview participants：**

Table S1 Sociodemographic characteristics of older people (n = 7).

| Participant ID | Gender | Age | Educational level | Marital status | Length of stay in the nursing home  (years) |
| --- | --- | --- | --- | --- | --- |
| A1 | Female | 82 | Primary school | Widowed | 5 |
| A2 | Male | 79 | Junior high school | Widowed | 1 |
| A3 | Female | 88 | Junior college | Widowed | 5 |
| A4 | Female | 87 | Junior college | Widowed | 5 |
| A5 | Male | 87 | Junior college | Married | 5 |
| A6 | Female | 80 | Junior high school | Widowed | 4 |
| A7 | Female | 82 | Junior high school | Married | 3 |

Table S2 Sociodemographic characteristics of care workers (n = 8).

| Participant ID | Gender | Age | Educational level | Years of experience in the nursing home | Professional qualification level |
| --- | --- | --- | --- | --- | --- |
| B1 | Female | 42 | Primary school | 2 | Junior level |
| B2 | Female | 41 | Primary school | 4 | Junior level |
| B3 | Female | 40 | Junior high school | 5 | Intermediate level |
| B4 | Female | 27 | Junior college | 4 | Senior level |
| B5 | Female | 45 | Primary school | 3 | Junior level |
| B6 | Female | 41 | Primary school | 3 | Junior level |
| B7 | Female | 45 | Primary school | 2 | Junior level |
| B8 | Male | 44 | Junior high school | 4 | Senior level |

Table S3 Sociodemographic characteristics of nurses (n = 5).

| Participant ID | Gender | Age | Educational level | Years of experience in the nursing home | Professional title |
| --- | --- | --- | --- | --- | --- |
| C1 | Female | 30 | Junior college | 6 | Nurse (intermediate professional title) |
| C2 | Female | 27 | Junior college | 3 | Nurse (intermediate professional title) |
| C3 | Female | 29 | Junior college | 4 | Nurse (intermediate professional title) |
| C4 | Male | 26 | Junior college | 2 | Nurse (intermediate professional title) |
| C5 | Female | 27 | Junior college | 3 | Nurse (intermediate professional title) |

*Note*：Professional titles refer to the standardized nursing professional title system in China.

Table S4 Sociodemographic characteristics of nursing managers (n = 6).

| Participant ID | Gender | Age | Educational level | Professional title | Position | Years of nursing experience | Years of managerial experience in the nursing home |
| --- | --- | --- | --- | --- | --- | --- | --- |
| D1 | Female | 40 | Junior college | Senior nurse | Head nurse | 16 | 2 |
| D2 | Female | 38 | Junior college | Senior nurse | Head nurse | 14 | 2 |
| D3 | Female | 41 | Junior college | Senior nurse | Head nurse | 17 | 5 |
| D4 | Female | 40 | Junior college | Senior nurse | Head nurse | 16 | 3 |
| D5 | Female | 39 | Junior college | Senior nurse | Head nurse | 15 | 3 |
| D6 | Female | 48 | Bachelor’s degree | Associate chief nurse | Director of the nursing department | 16 | 4 |
