# Supplementary material for: Construction of an evaluation indicator system for humanistic care quality in nursing homes
Source: BMC Geriatr. 2026 May 8;26:899. doi: 10.1186/s12877-026-07623-3 (PMC13330427; doi:10.1186/s12877-026-07623-3)
Supplement: Supplementary file 3 — Supplementary Material 3. [file 12877_2026_7623_MOESM3_ESM.docx]

**Round 1:**

Table S1 The revision of the secondary-level indicators of the evaluation index system.

| **Initial items** | **Outcome** | **Final items** |
| --- | --- | --- |
| I-2 Elderly care human resources | modified | Nursing workforce development |
| II-1 Caring environment |  | II-1 should be classified under structure quality |
| II-6 Communication and interaction |  | Communication and comfort |
| II-7 Personalized caring |  | Appreciation of individual uniqueness |
| II-8 Respect、encouragement |  | Respect and encouragement |
| III-1 Older people and their family members |  | Evaluation by older people and their family members (This indicator was then further divided into:“Evaluation by older people” and “Evaluation by family members” ) |
| III-2 Elderly care staff |  | Evaluation by nursing staff |
| II-2 Meeting daily living care needs | merged | Meeting basic daily living care needs |
| II-3 Meeting physical health care needs |  |  |
| II-4 Meeting spiritual and cultural care needs |  |  |
| I-3 Older people and their family members | deleted |  |
| III-3 Nursing homes |  |  |

Table S2 The revision of the tertiary-level indicators of the evaluation index system.

| **Initial items** | **Outcome** | **Final items** |
| --- | --- | --- |
| I-1-1 Establishment of a humanistic caring quality management group in nursing homes | modified | Establishment of secondary- or tertiary-level humanistic caring quality management groups |
| I-1-2 Development, implementation, and monitoring of humanistic caring policies and institutional regulations in nursing homes |  | Development of humanistic caring work procedures, practice standards, and inspection criteria in nursing homes |
| I-1-3 Development, implementation, and documentation of training programs related to humanistic caring theories and skills |  | Provision of training on humanistic caring knowledge and skills |
| I-1-4 Assessment of theory and skills related to humanistic care and the situation of continuous improvement |  | Assessment and continuous improvement of humanistic caring knowledge and skills |
| I-2-1 Appropriate ratio of elderly care nursing staff to older people |  | Allocation of elderly care nursing staff based on older people’s functional assessment levels and care needs |
| I-2-3 Fostering professional identity and a sense of honor among elderly care nursing staff and encouraging individual career development |  | Establishment of incentive mechanisms for humanistic caring and assurance of nursing staff welfare benefits |
| I-2-5 Cooperation and mutual support among institutional staff with a harmonious working atmosphere |  | Implement activities to support and care for nursing staff |
| II-1-3 Provision of leisure and recreational activity spaces and materials with regular cleaning and disinfection |  | Diverse and well-equipped activity spaces |
| II-4-6 Encouragement and support for older people to participate in mutual-aid elderly volunteer services and contribute their experience |  | Encouragement of older people to participate in activities within their capabilities to achieve self-worth |
| II-4-8 Provision of necessary safety protection measures for older people during activities |  | Implement safety assurance measures |
| II-5-3 Provision of life-and-death education for older people and their family members to promote appropriate perspectives on life and death |  | Provision of palliative and end-of-life care for terminally ill older people |
| II-5-4 Provision of opportunities for interaction and communication among older people to foster friendly and harmonious relationships |  | Attention to and coordination of interpersonal relationships between older people |
| II-5-5 Assistance for older people in maintaining contact with family members and encouragement of family visits and involvement in care |  | Assistance for older people in maintaining contact with family members |
| II-5-6 Provision of social volunteer services to meet older people’s needs for emotional companionship |  | Implementation of volunteer services supporting older people |
| II-8-4 Respect for older people’s right to be informed, with timely provision of information related to activities, care, and services |  | Respect for older people’s right to be informed, and provide information related to care, activities and other services |
| II-8-5 Avoidance of infantilizing older people, such as using childish language or assigning nicknames |  | Respect for older people’s dignity and avoidance of infantilizing treatment of cognitively intact older people |
| II-8-6 Protection of older people’s privacy during care |  | Respect for and protection of older people’s physical privacy and personal information privacy |
| II-8-9 Encouragement of older people to engage in self-care and adopt health-promoting behaviors |  | Encouragement of self-care among older people, with emphasis on health awareness and proactive adoption of healthy behaviors |
| II-8-11 Recognition of older people’s performance and life experiences, providing confidence and hope |  | Appreciation and affirmation of older people’s past achievements and current performance |
| III-1-1 Satisfaction of older people and their family members with humanistic caring services in nursing homes |  | III-1-1 was split into “Satisfaction of older people with humanistic caring” and “Satisfaction of family members with humanistic caring |
| III-1-2 Quality of life of older people |  | Evaluation of older people’s quality of life |
| III-2-1 Job satisfaction of elderly care nursing staff |  | Self-evaluation of humanistic caring quality by nursing staff |
| III-2-2 Professional identity and sense of belonging among elderly care nursing staff |  | Work well-being index of nursing staff |
| “II-1-1 A warm and harmonious public environment with a strong culture of respect for older people” and “II-1-2 Spacious, comfortable, clean, and tidy living spaces for older people” | merged | Warm and comfortable living environment |
| “II-1-4 A well-designed signage and wayfinding system that is clear and easy to understand (e.g., friendly reminders and public safety signs)” and “II-1-5 Provision of age-friendly assistive devices and facilities” |  | Ensure environmental safety and privacy protection |
| “II-2-1 Regular assessment of older people’s dietary and nutritional status and needs,” “II-2-2 Provision of nutritionally balanced meals that meet cultural customs and food safety requirements and are suitable for older people,” “II-2-3 Provision of meal assistance services to ensure safety during dining,” “II-2-4 Assessment of older people’s sleep status and needs and provision of a favorable sleep environment,” “II-2-5 Assessment of older people’s personal hygiene needs and assistance with personal care (e.g., bathing),” “II-3-1 Assessment of muscle strength and functional ability and arrangement of and assistance with rehabilitation training,” and “II-3-2 Dynamic assessment of physical health status and provision of medical assistance services (e.g., emergency care, referral, and accompaniment)” |  | Provide basic daily living care |
| “II-3-4 Regular provision of health education on wellness and rehabilitation” and “II-4-7 Organization of physical fitness and exercise activities for older people” |  | Provide health care services |
| “II-4-1 Assessment of older people’s needs for spiritual and cultural services,” “II-4-2 Provision of educational courses for older people,” “II-4-3 Regular organization of outdoor leisure activities (e.g., spring and autumn outings),” and “II-4-4 Regular organization of activities such as painting, singing, movie watching, chess playing, and handicrafts” |  | Provision of cultural and recreational activities |
| “II-6-1 Nursing staff dress appropriately and behave professionally,” “II-6-2 Appropriate forms of address and communication in accordance with service standards,” and “II-6-3 Patient listening and timely responses” |  | Humanistic communication in care |
| “II-5-1 Dynamic assessment of older people’s psychological status, with particular attention to newly admitted and end-of-life residents, and timely communication with older people and their family members when abnormalities are identified,” and “II-5-2 Provision of emotional support and psychological counseling services as needed” |  | Assessment of psychological status and provision of psychological support |
| “II-8-2 Respect for older people’s autonomy in personal decision-making” and “II-8-3 Respect for older people’s individuality and preservation of their original living habits whenever possible” |  | Respect for older people’s personal preferences and encouragement of shared care planning |
| “II-6-5 Follow-up care for older people after leaving the facility (e.g., telephone follow-up regarding health status)” and “II-7-5 Celebrating older people’s birthdays and offering warm birthday wishes” |  | Build harmonious interpersonal relationships between nursing staff and older people |
| I-2-2 Professional qualifications of elderly care nursing staff | deleted |  |
| I-2-3 Elderly care nursing staff adhere to professional ethics and demonstrate good professional conduct |  |  |
| I-3-1 Establishment of personal and family information records (e.g., family background, living habits) |  |  |
| I-3-2 Regular assessment of the caring needs of older people and their family members |  |  |
| II-1-6 Clear zoning of self-care, semi-dependent, and dependent areas |  |  |
| II-1-7 Convenient transportation and ease of mobility |  |  |
| II-3-3 Regular assessment of older people’s self-care ability and dynamic adjustment of care levels |  |  |
| II-4-5 Provision of a reading room (or reading corner) with books, newspapers, and magazines |  |  |
| II-5-7 Active engagement in public outreach to attract social support and participation in elderly care services |  |  |
| II-6-4 Good service attitude and equal treatment of all older people |  |  |
| II-6-6 Establishment of a trusting and caring relationship with older people and their family members |  |  |
| II-7-1 Encouraging older people to express their own ideas, wishes, and needs |  |  |
| II-7-2 Provision of and assistance with personalized activities based on a comprehensive consideration of older people’s characteristics |  |  |
| II-7-3 Reasonable allocation of rooms and beds according to older people’s living habits and self-care ability |  |  |
| II-7-4 Allowing older people to decorate their rooms according to personal preferences |  |  |
| II-8-7 Arranging same-gender nursing staff for older people whenever possible |  |  |
| II-8-8 Demonstration of empathy and respect for older people’s feelings during service provision |  |  |
| II-8-10 Encouraging older people to participate in nursing care decision-making |  |  |
| III-3-1 Self-evaluation of the overall quality of humanistic caring by institutional managers |  |  |
| III-3-2 Self-evaluation of the reputation and visibility of the nursing home |  |  |
|  | added | Establishment of appropriate values for elderly care services and a humanistic caring philosophy |
|  |  | Establishment of a volunteer service organization and management system |
|  |  | Provision of nostalgic emotional value |
|  |  | Support for the realization of self-worth |
|  |  | Respect for older people’s right to supervision and encouragement of their participation in institutional management |
|  |  | Attention to older people’s life background and worldview |

**Round 2:**

Table S3 The revision of the secondary-level indicators of the evaluation index system.

| **Initial items** | **Outcome** | **Final items** |
| --- | --- | --- |
| II-3 Appreciation of individual uniqueness | modified | Appreciation of older people’s self-worth |
| III-1 Evaluation by older people | merged | Evaluation by older people and their family members |
| III-2 Evaluation by family members |  |  |

Table S4 The revision of the tertiary-level indicators of the evaluation index system.

| **Initial items** | **Outcome** | **Final items** |
| --- | --- | --- |
| I-1-1 Establishment of secondary- or tertiary-level humanistic caring quality management groups | modified | Establishment of humanistic caring quality management groups at all levels |
| I-1-3 Establishment of appropriate values for elderly care services and a humanistic caring philosophy |  | I-1-3 would be more appropriately classified under process quality |
| I-2-3 Implementation of staff-care activities and provision of a comfortable working environment for nursing staff |  | Implementation of staff-care activities for nursing staff. |
| I-3-3 Diverse and well-equipped activity spaces |  | Appropriate configuration of various functional spaces |
| II-2-3 Respect for older people’s dignity and avoidance of infantilizing treatment of cognitively intact older people |  | Respect older people’s personal dignity and avoid infantilizing cognitively intact older people |
| II-4-4 Provision of cultural and recreational activities |  | Provide leisure and recreational activities |
| II-5-2 Attention to and coordination of interpersonal relationships between older people |  | Attend to and coordinate interpersonal relationships among older people |
| II-2-4 Respect for older people’s right to be informed, and provide information related to care, activities and other services | merged | Respect for older people’s right to be informed and encouragement of shared care planning |
| II-2-6 Respect for older people’s personal preferences and encouragement of shared care planning |  |  |
| II-5-5 Provide palliative care for older people at the end of life | deleted |  |
