# Supplementary material for: Construction of an evaluation indicator system for humanistic care quality in nursing homes
Source: BMC Geriatr. 2026 May 8;26:899. doi: 10.1186/s12877-026-07623-3 (PMC13330427; doi:10.1186/s12877-026-07623-3)
Supplement: Supplementary file 4 — Supplementary Material 4. [file 12877_2026_7623_MOESM4_ESM.docx]

**1. Judgment matrix, weights, and consistency ratio (CR) of primary-level indicators.**

Table S1 Judgment matrix, weights, and consistency ratio (CR) of primary-level indicators.

| Indicators | I-1 Structure | I-2 Process | I-3 Outcome | *Wi* | *λmax* | *CR* |
| --- | --- | --- | --- | --- | --- | --- |
| I-1 Structure | 1 | 0.5 | 2 | 0.3108 | 3.0536 | 0.0516 |
| I-2 Process | 2 | 1 | 2 | 0.4934 |  |  |
| I-3 Outcome | 0.5 | 0.5 | 1 | 0.1958 |  |  |

**2. Judgment matrix, weights, and consistency test of secondary-level indicators.**

Table S2 Judgment matrix, weights, and consistency test of secondary-level indicators.

| I-1 Structure | II-1 | II-2 | II-3 | *Wi* | *λmax* | *CR* |
| --- | --- | --- | --- | --- | --- | --- |
| II-1 | 1 | 2 | 4 | 0.5584 | 3.0183 | 0.0176 |
| II-2 | 0.5 | 1 | 3 | 0.3196 |  |  |
| II-3 | 0.25 | 0.3333 | 1 | 0.1220 |  |  |

Table S3 Judgment matrix, weights, and consistency test of secondary-level indicators.

| I-2 Process | II-4 | II-5 | II-6 | II-7 | II-8 | *Wi* | *λmax* | *CR* |
| --- | --- | --- | --- | --- | --- | --- | --- | --- |
| II-4 | 1 | 1 | 2 | 2 | 4 | 0.2962 | 5.1363 | 0.0304 |
| II-5 | 1 | 1 | 2 | 2 | 4 | 0.2962 |  |  |
| II-6 | 0.5 | 0.5 | 1 | 0.25 | 4 | 0.1509 |  |  |
| II-7 | 0.5 | 0.5 | 2 | 1 | 4 | 0.1997 |  |  |
| II-8 | 0.25 | 0.25 | 0.25 | 0.25 | 1 | 0.0570 |  |  |

Table S4 Judgment matrix, weights, and consistency test of secondary-level indicators.

| I-3 Outcome | II-9 | II-10 | *Wi* | *λmax* | *CR* |
| --- | --- | --- | --- | --- | --- |
| II-9 | 1 | 3 | 0.7500 | 0.0000 | 2.0000 |
| II-10 | 0.3333 | 1 | 0.2500 |  |  |

1. **Judgment matrix, weights, and consistency test of tertiary-level indicators.**

Table S5 Judgment matrix, weights, and consistency test of tertiary-level indicators.

| II-1 Humanistic care system | III-1 | III-2 | III-3 | III-4 | *Wi* | *λmax* | *CR* |
| --- | --- | --- | --- | --- | --- | --- | --- |
| III-1 | 1 | 0.3333 | 0.5 | 2 | 0.1636 | 4.0710 | 0.0266 |
| III-2 | 3 | 1 | 2 | 3 | 0.4476 |  |  |
| III-3 | 2 | 0.5 | 1 | 3 | 0.2829 |  |  |
| III-4 | 0.5 | 0.3333 | 0.3333 | 1 | 0.1059 |  |  |

Table S6 Judgment matrix, weights, and consistency test of tertiary-level indicators.

| II-2 Nursing workforce building | III-5 | III-6 | III-7 | III-8 | III-9 | III-10 | *Wi* | *λmax* | *CR* |
| --- | --- | --- | --- | --- | --- | --- | --- | --- | --- |
| III-5 | 1 | 0.25 | 0.25 | 0.3333 | 0.25 | 0.3333 | 0.0483 | 6.2472 | 0.0392 |
| III-6 | 4 | 1 | 2 | 3 | 2 | 3 | 0.3153 |  |  |
| III-7 | 4 | 0.5 | 1 | 3 | 0.5 | 3 | 0.1983 |  |  |
| III-8 | 3 | 0.3333 | 0.3333 | 1 | 0.3333 | 1 | 0.0941 |  |  |
| III-9 | 4 | 0.5 | 2 | 3 | 1 | 3 | 0.25 |  |  |
| III-10 | 3 | 0.3333 | 0.3333 | 1 | 0.3333 | 1 | 0.0941 |  |  |

Table S7 Judgment matrix, weights, and consistency test of tertiary-level indicators.

| II-3 Caring environment | III-11 | III-12 | III-13 | III-14 | *Wi* | *λmax* | *CR* |
| --- | --- | --- | --- | --- | --- | --- | --- |
| III-11 | 1 | 2 | 1 | 2 | 0.33 | 4.0606 | 0.0227 |
| III-12 | 0.5 | 1 | 0.5 | 2 | 0.1996 |  |  |
| III-13 | 1 | 2 | 1 | 2 | 0.33 |  |  |
| III-14 | 0.5 | 0.5 | 0.5 | 1 | 0.1404 |  |  |

Table S8 Judgment matrix, weights, and consistency test of tertiary-level indicators.

| II-4 Meeting basic life care needs | III-15 | III-16 | III-17 | III-18 | *Wi* | *λmax* | *CR* |
| --- | --- | --- | --- | --- | --- | --- | --- |
| III-15 | 1 | 0.3333 | 2 | 2 | 0.231 | 4.1213 | 0.0454 |
| III-16 | 3 | 1 | 3 | 3 | 0.4901 |  |  |
| III-17 | 0.5 | 0.3333 | 1 | 2 | 0.1634 |  |  |
| III-18 | 0.5 | 0.3333 | 0.5 | 1 | 0.1155 |  |  |

Table S9 Judgment matrix, weights, and consistency test of tertiary-level indicators.

| II-5 Communication and comfort | III-19 | III-20 | *Wi* | *λmax* | *CR* |
| --- | --- | --- | --- | --- | --- |
| III-19 | 1 | 2 | 0.6667 | 0.0000 | 2.0000 |
| III-20 | 0.5 | 1 | 0.3333 |  |  |

Table S10 Judgment matrix, weights, and consistency test of tertiary-level indicators.

| II-6 Meeting care needs for love and belonging | III-21 | III-22 | III-23 | III-24 | *Wi* | *λmax* | *CR* |
| --- | --- | --- | --- | --- | --- | --- | --- |
| III-21 | 1 | 0.3333 | 1 | 0.3333 | 0.1222 | 4.0606 | 0.0227 |
| III-22 | 3 | 1 | 3 | 0.5 | 0.3121 |  |  |
| III-23 | 1 | 0.3333 | 1 | 0.3333 | 0.1222 |  |  |
| III-24 | 3 | 2 | 3 | 1 | 0.4435 |  |  |

Table S11 Judgment matrix, weights, and consistency test of tertiary-level indicators.

| II-7 Respect and encouragement | III-25 | III-26 | III-27 | III-28 | III-29 | III-30 | *Wi* | *λmax* | *CR* |
| --- | --- | --- | --- | --- | --- | --- | --- | --- | --- |
| III-25 | 1 | 0.3333 | 3 | 3 | 0.5 | 3 | 0.1824 | 6.3224 | 0.0512 |
| III-26 | 3 | 1 | 3 | 3 | 2 | 3 | 0.3318 |  |  |
| III-27 | 0.3333 | 0.3333 | 1 | 0.5 | 0.3333 | 0.5 | 0.0643 |  |  |
| III-28 | 0.3333 | 0.3333 | 2 | 1 | 0.3333 | 2 | 0.1017 |  |  |
| III-29 | 2 | 0.5 | 3 | 3 | 1 | 3 | 0.2389 |  |  |
| III-30 | 0.3333 | 0.3333 | 2 | 0.5 | 0.3333 | 1 | 0.0809 |  |  |

Table S12 Judgment matrix, weights, and consistency test of tertiary-level indicators.

| II-8 Appreciating the self-worth of older people | III-31 | III-32 | III-33 | *Wi* | *λmax* | *CR* |
| --- | --- | --- | --- | --- | --- | --- |
| III-31 | 1 | 1 | 0.5 | 0.25 | 3.0000 | 0.0000 |
| III-32 | 1 | 1 | 0.5 | 0.25 |  |  |
| III-33 | 2 | 2 | 1 | 0.5 |  |  |

Table S13 Judgment matrix, weights, and consistency test of tertiary-level indicators.

| II-9 Older people’s and family members’ evaluation | III-34 | III-35 | III-36 | *Wi* | *λmax* | *CR* |
| --- | --- | --- | --- | --- | --- | --- |
| III-34 | 1 | 3 | 4 | 0.625 | 3.0183 | 0.0176 |
| III-35 | 0.3333 | 1 | 2 | 0.2385 |  |  |
| III-36 | 0.25 | 0.5 | 1 | 0.1365 |  |  |

Table S14 Judgment matrix, weights, and consistency test of tertiary-level indicators.

| II-10 Evaluation by nursing staff | III-37 | III-38 | *Wi* | *λmax* | *CR* |
| --- | --- | --- | --- | --- | --- |
| III-37 | 1 | 4 | 0.8 | 2.0000 | 0.0000 |
| III-38 | 0.25 | 1 | 0.2 |  |  |

**4.Weights of indicators at all levels and consistency tests**

Table S15 Weights of indicators at all levels and consistency tests

| **Hierarchical level** | **Indicators** | **Initial weight** | **Composite weight** | ***CR*** | ***λmax*** |
| --- | --- | --- | --- | --- | --- |
| Primary-level | I-1 Structure | 0.3108 | 0.3108 | 0.0516 | 3.0536 |
|  | I-2 Process | 0.4934 | 0.4934 |  |  |
|  | I-3 Outcome | 0.1958 | 0.1958 |  |  |
| Secondary-level | II-1 Humanistic care system | 0.5584 | 0.1736 | 0.0176 | 3.0183 |
|  | II-2 Nursing workforce development | 0.3196 | 0.0993 |  |  |
|  | II-3 Caring environment | 0.1220 | 0.0379 |  |  |
|  | II-4 Meeting basic daily living care needs | 0.2962 | 0.1462 | 0.0304 | 5.1363 |
|  | II-5 Communication and comfort | 0.2962 | 0.1462 |  |  |
|  | II-6 Meeting care needs for love and belonging | 0.1509 | 0.0744 |  |  |
|  | II-7 Respect and encouragement | 0.1997 | 0.0985 |  |  |
|  | II-8 Appreciation of older people’s self-worth | 0.0570 | 0.0281 |  |  |
|  | II-9 Evaluation by older people and their family members | 0.7500 | 0.1469 | 0.0000 | 2.0000 |
|  | II-10 Evaluation by nursing staff | 0.2500 | 0.0490 |  |  |
| Tertiary-level | III-1 Establishment of humanistic caring quality management groups at all levels | 0.1636 | 0.0284 | 0.0266 | 4.0710 |
|  | III-2 Develop humanistic caring workflows, practice guidelines, and inspection standards | 0.4476 | 0.0777 |  |  |
|  | III-3 Rationally allocate the number of elderly care nursing staff based on older people’s functional assessment levels and care needs | 0.2829 | 0.0491 |  |  |
|  | III-4 Establish an organizational and management system for volunteer services | 0.1059 | 0.0184 |  |  |
|  | III-5 Establish appropriate values for elderly care services and a humanistic caring philosophy | 0.0483 | 0.0048 | 0.0392 | 6.2472 |
|  | III-6 Provide training in humanistic caring knowledge and skills | 0.3153 | 0.0313 |  |  |
|  | III-7 Conduct assessment and continuous improvement of humanistic caring knowledge and skills | 0.1983 | 0.0197 |  |  |
|  | III-8 Establish incentive mechanisms for humanistic caring practices and safeguard nursing staff welfare and benefits | 0.0941 | 0.0093 |  |  |
|  | III-9 Implement activities to support and care for nursing staff | 0.2500 | 0.0248 |  |  |
|  | III-10 Foster a harmonious and mutually supportive work environment | 0.0941 | 0.0093 |  |  |
|  | III-11 Create a warm and comfortable living environment | 0.3300 | 0.0125 | 0.0227 | 4.0606 |
|  | III-12 Ensure environmental safety and privacy protections | 0.1996 | 0.0076 |  |  |
|  | III-13 Appropriate configuration of various functional spaces | 0.3300 | 0.0125 |  |  |
|  | III-14 Provide nostalgic emotional value | 0.1404 | 0.0053 |  |  |
|  | III-15 Provide basic daily living care | 0.2310 | 0.0338 | 0.0454 | 4.1213 |
|  | III16 Implement safety assurance measures | 0.4901 | 0.0716 |  |  |
|  | III-17 Provide health care services | 0.1634 | 0.0239 |  |  |
|  | III-18 Provide leisure and recreational activities | 0.1155 | 0.0169 |  |  |
|  | III-19 Emphasize humanistic caring in communication | 0.6667 | 0.0974 | 0.0000 | 2.0000 |
|  | III-20 Assess older people’s psychological status and provide psychological comfort | 0.3333 | 0.0487 |  |  |
|  | III-21 Build harmonious interpersonal relationships between nursing staff and older people | 0.1222 | 0.0091 | 0.0227 | 4.0606 |
|  | III-22 Attend to and coordinate interpersonal relationships among older people | 0.3121 | 0.0232 |  |  |
|  | III-23 Assisting older people in maintaining contact with their families | 0.1222 | 0.0091 |  |  |
|  | III-24 Carry out volunteer services to support older people | 0.4435 | 0.0330 |  |  |
|  | III-25 Respect older people’s cultural customs and religious beliefs | 0.1824 | 0.0180 | 0.0512 | 6.3224 |
|  | III-26 Respect and protect older people’s bodily privacy and personal information privacy | 0.3318 | 0.0327 |  |  |
|  | III-27 Respect older people’s personal dignity and avoid infantilizing cognitively intact older people | 0.0643 | 0.0063 |  |  |
|  | III-28 Respect for older people’s right to be informed and encouragement of shared care planning | 0.1017 | 0.0100 |  |  |
|  | III-29 Respect older people’s right to supervision and encourage shared participation in institutional management | 0.2389 | 0.0235 |  |  |
|  | III-30 Encourage self-care among older people, emphasizing attention to personal health and the adoption of healthy behaviors | 0.0809 | 0.0080 |  |  |
|  | III-31 Pay attention to older people’s life backgrounds and their views on life, the world, and values | 0.2500 | 0.0070 | 0.0000 | 3.0000 |
|  | III-32 Appreciate and affirm older people’s past achievements and current performance | 0.2500 | 0.0070 |  |  |
|  | III-33 Identify and cultivate older people’s interests and strengths | 0.5000 | 0.0141 |  |  |
|  | III-34 Older people’s satisfaction with humanistic caring | 0.6250 | 0.0918 | 0.0176 | 3.0183 |
|  | III-35 Evaluation of older people’s quality of life | 0.2385 | 0.0350 |  |  |
|  | III-36 Family members’ satisfaction with humanistic care | 0.1365 | 0.0200 |  |  |
|  | III-37 Nursing staff’s self-evaluation of humanistic caring quality | 0.8000 | 0.0392 | 0.0000 | 2.0000 |
|  | III-38 Work well-being index of nursing staff | 0.2000 | 0.0098 |  |  |

**5.The importance scores and coefficient of variation of the humanistic caring quality indicator system for the older people in nursing homes**

Table S16 The importance scores and coefficient of variation of the humanistic caring quality indicator system for the older people in nursing homes

| **Indicators** | **Importance value**  **(**$\bar{\text{x}}\text{±}\text{s}$**)** | **coefficient of variation (%)** |
| --- | --- | --- |
| **1 Structure** | 4.97±0.18 | 3.56 |
| **1.1 Humanistic care system** | 5.00±0.00 | 0.00 |
| 1.1.1 Establishment of humanistic caring quality management groups at all levels | 4.69±0.47 | 10.05 |
| 1.1.2 Develop humanistic caring workflows, practice guidelines, and inspection standards | 4.97±0.18 | 3.56 |
| 1.1.3 Rationally allocate the number of elderly care nursing staff based on older people’s functional assessment levels and care needs | 4.94±0.25 | 4.98 |
| 1.1.4 Establish an organizational and management system for volunteer services | 4.63±0.55 | 11.97 |
| **1.2 Nursing workforce development** | 4.97±0.18 | 3.56 |
| 1.2.1 Establish appropriate values for elderly care services and a humanistic caring philosophy | 4.31±0.47 | 10.92 |
| 1.2.2 Provide training in humanistic caring knowledge and skills | 4.97±0.18 | 3.56 |
| 1.2.3 Conduct assessment and continuous improvement of humanistic caring knowledge and skills | 4.88±0.34 | 6.89 |
| 1.2.4 Establish incentive mechanisms for humanistic caring practices and safeguard nursing staff welfare and benefits | 4.59±0.50 | 10.86 |
| 1.2.5 Implement activities to support and care for nursing staff | 4.91±0.30 | 6.04 |
| 1.2.6 Foster a harmonious and mutually supportive work environment | 4.59±0.50 | 10.86 |
| **1.3 Caring environment** | 4.47±0.51 | 11.35 |
| 1.3.1 Create a warm and comfortable living environment | 4.69±0.47 | 10.05 |
| 1.3.2 Ensure environmental safety and privacy protections | 4.56±0.50 | 11.05 |
| 1.3.3 Appropriate configuration of various functional spaces | 4.69±0.54 | 11.41 |
| 1.3.4 Provide nostalgic emotional value | 4.50±0.57 | 12.62 |
| **2 Process** | 5.00±0.00 | 0.00 |
| **2.1 Meeting basic daily living care needs** | 4.97±0.18 | 3.56 |
| 2.1.1 Provide basic daily living care | 4.63±0.55 | 11.97 |
| 2.1.2 Implement safety assurance measures | 4.91±0.39 | 7.95 |
| 2.1.3 Provide health care services | 4.59±0.56 | 12.19 |
| 2.1.4 Provide leisure and recreational activities | 4.50±0.57 | 12.62 |
| **2.2 Communication and comfort** | 4.97±0.18 | 3.56 |
| 2.2.1 Emphasize humanistic caring in communication | 4.94±0.25 | 4.98 |
| 2.2.2 Assess older people’s psychological status and provide psychological comfort | 4.88±0.42 | 8.64 |
| **2.3 Meeting care needs for love and belonging** | 4.91±0.30 | 6.04 |
| 2.3.1 Build harmonious interpersonal relationships between nursing staff and older people | 4.53±0.57 | 12.51 |
| 2.3.2 Attend to and coordinate interpersonal relationships among older people | 4.88±0.42 | 8.64 |
| 2.3.3 Assisting older people in maintaining contact with their families | 4.53±0.57 | 12.51 |
| 2.3.4 Carry out volunteer services to support older people | 4.94±0.25 | 4.98 |
| **2.4 Respect and encouragement** | 4.94±0.25 | 4.98 |
| 2.4.1 Respect older people’s cultural customs and religious beliefs | 4.81±0.40 | 8.24 |
| 2.4.2 Respect and protect older people’s bodily privacy and personal information privacy | 4.91±0.30 | 6.04 |
| 2.4.3 Respect older people’s personal dignity and avoid infantilizing cognitively intact older people | 4.44±0.56 | 12.72 |
| 2.4.4 Respect for older people’s right to be informed and encouragement of shared care planning | 4.53±0.51 | 11.19 |
| 2.4.5 Respect older people’s right to supervision and encourage shared participation in institutional management | 4.88±0.34 | 6.89 |
| 2.4.6 Encourage self-care among older people, emphasizing attention to personal health and the adoption of healthy behaviors | 4.50±0.51 | 11.29 |
| **2.5 Appreciation of older people’s self-worth** | 4.34±0.55 | 12.55 |
| 2.5.1 Pay attention to older people’s life backgrounds and their views on life, the world, and values | 4.50±0.51 | 11.29 |
| 2.5.2 Appreciate and affirm older people’s past achievements and current performance | 4.50±0.57 | 12.62 |
| 2.5.3 Identify and cultivate older people’s interests and strengths | 4.72±0.52 | 11.08 |
| **3 Outcome** | 4.94±0.25 | 4.98 |
| **3.1 Evaluation by older people and their family members** | 4.97±0.18 | 3.56 |
| 3.1.1 Older people’s satisfaction with humanistic caring | 4.88±0.34 | 6.89 |
| 3.1.2 Evaluation of older people’s quality of life | 4.53±0.51 | 11.19 |
| 3.1.3 Family members’ satisfaction with humanistic care | 4.28±0.89 | 20.75 |
| **3.2 Evaluation by nursing staff** | 4.59±0.56 | 12.19 |
| 3.2.1 Nursing staff’s self-evaluation of humanistic caring quality | 4.97±0.18 | 3.56 |
| 3.2.2 Work well-being index of nursing staff | 4.28±0.89 | 20.75 |
