# Supplementary material for: Construction of an evaluation indicator system for humanistic care quality in nursing homes
Source: BMC Geriatr. 2026 May 8;26:899. doi: 10.1186/s12877-026-07623-3 (PMC13330427; doi:10.1186/s12877-026-07623-3)
Supplement: Supplementary file 5 — Supplementary Material 5. [file 12877_2026_7623_MOESM5_ESM.docx]

第一轮专家函询表

养老机构人文关怀照护质量评价指标体系

专家函询（第一轮）

尊敬的专家：

您好！非常感谢您在百忙之中参与本次函询。

我们是浙江省浙江中医药大学护理学院沈勤教授研究团队，目前正在进行《养老机构人文关怀照护质量评价指标体系的构建》的课题研究。鉴于您在本学科的学术造诣和厚重的专业底蕴，且工作经验丰富，我们非常荣幸地邀请您作为本课题的咨询专家。请您按顺序逐项填写，千万不要遗漏。您的意见和建议将作为我们构建养老机构人文关怀照护质量评价指标体系的重要依据。

本问卷内容包含三个部分：第一部分为课题背景；第二部分为养老机构人文关怀照护质量评价指标构建的函询问卷；第三部分为专家的个人基本情况。此咨询表以匿名形式函询，本课题组将对您的资料进行严格的保密。为保证问卷的准确性及效率性，敬请您在收到咨询表**2周内**完成。希望您能提出宝贵的意见，衷心感谢您对本课题的支持与帮助！

谨祝：身体健康，万事如意！

第一部分 课题背景简介

护理质量评价贯穿于护理活动的整个过程，是管理组织根据相关的护理管理标准，对护理活动进行调查、分析，客观评价质量差异的过程。养老机构已成为我国养老模式的重要组成部分，但是经过我们前期文献回顾、质性研究及实地调研发现，整体上缺乏对养老机构人文关怀照护的关注。现有的养老机构护理模式普遍较为刻板，多以保障老年人的安全和提供基础护理服务为主，老年人的精神文化生活和心理健康等方面的需求都不同程度地被忽视。且研究较局限于老年人关怀需求、关怀措施及影响因素研究，国内现有的评价标准多以机构为中心，少以老人为中心，尚缺乏一套系统化的、切实可行的养老机构人文关怀照护质量指标体系。因此，本研究进行“养老机构人文关怀照护质量评价指标体系”的研究，为日后养老机构人文关怀照护质量评价及持续改进提供参考依据。

质量关怀模式（quality caring model，QCM）是美国Joanne Duffy博士在Watson人性关怀科学理论的基础上，将八个关怀因素结合Donabedian的结构一过程一结果三维质量理论构建起来的一种模式。她认为在现代医疗保健的现实中应保存护理的本质，推崇护理最深层的价值即关怀，提出八个关怀因素，包括细心的安慰、基本的人类需求、鼓励的态度、共同解决问题、归属的需要、治愈性环境、人类的尊重和欣赏独特意义。本研究将以该模式作为理论框架，从结构、过程、结局三个维度及八个关怀因素出发，构建养老机构人文关怀照护质量评价指标体系。

马斯洛需求层次理论是由美国著名学者马斯洛提出，他认为人类的需求按其属性从低到高依次分为生理需求、安全需求、爱与归属需求、自尊与尊重的需求、自我实现的需求五个层次。本研究基于马斯洛需求层次理论内涵，分析老年人在养老机构人文关怀照护服务中的需求。

本研究在检索国内外文献的基础上，结合养老机构老年人、护士、护理管理者的质性访谈，形成初级指标，其中一级指标3个，二级指标14个，三级指标70个。敬请您对初步形成的指标框架提出宝贵的意见。

第二部分 养老机构人文关怀照护质量评价指标

填表说明：

1、请您根据个人经验和理解，判断每个评价指标的重要性程度，在您认为每项内容对应的选项框内打“√”。本部分共有3个表格，分别为一级指标、二级指标、三级指标的判断。

2、若您认为评价指标在语言表述或内容等方面有问题，请在“修改意见”栏内修改；若您认为我们还有未考虑到的项目，请您在“需增加／删改指标”栏内添加，修改及增加的项目同样需要判断其重要程度，在相应栏内打“√”或将相应表格内的数值标红。

3、请勿空项或漏项，非常感谢您的支持与指导！

表1一级指标专家咨询表

| First-level | Definition | 重要性评分 | | | | | 修改意见 |
| --- | --- | --- | --- | --- | --- | --- | --- |
|  |  | 5 | 4 | 3 | 2 | 1 |  |
|  |  | 很重要 | 较重要 | 一般重要 | 不太重要 | 不重要 |  |
| I Structure | 构成人文关怀照护的基本条件和要素，如人员、培训情况、规章制度、硬件设备等。 |  |  |  |  |  |  |
| II Process | 护理人员根据老年人的状况及活动能力提供的服务活动及其他补充性活动。 |  |  |  |  |  |  |
| III Outcome | 人文关怀照护对老年人健康照护或结局的结果，也包括护理人员结局。 |  |  |  |  |  |  |
| 补充建议： |  | | | | | | |

对于表1，若您还有其他意见，请在这里填写：

表2二级指标咨询表

| First-level indicators | Second-level indicators | 很重要 | 较重要 | 一般  重要 | 不太重要 | 不重要 | 修改意见 |
| --- | --- | --- | --- | --- | --- | --- | --- |
|  |  | 5 | 4 | 3 | 2 | 1 |  |
| I Structure | I-1人文关怀照护系统 |  |  |  |  |  |  |
|  | I-2养老护理人力资源 |  |  |  |  |  |  |
|  | I-3老人及家属 |  |  |  |  |  |  |
|  | **需增加的指标：** |  |  |  |  |  |  |
| II Process | II-1关怀性环境 |  |  |  |  |  |  |
|  | II-2满足日常生活照护需求 |  |  |  |  |  |  |
|  | II-3满足身体健康照护需求 |  |  |  |  |  |  |
|  | II-4满足精神文化照护需求 |  |  |  |  |  |  |
|  | II-5满足爱与归属照护需求 |  |  |  |  |  |  |
|  | II-6沟通交往 |  |  |  |  |  |  |
|  | II-7个性化关怀 |  |  |  |  |  |  |
|  | II-8尊重、鼓励 |  |  |  |  |  |  |
|  | **需增加的指标：** |  |  |  |  |  |  |
| III Outcome | III-1 老人及家属 |  |  |  |  |  |  |
|  | III-2 养老护理人员 |  |  |  |  |  |  |
|  | III-3 养老机构 |  |  |  |  |  |  |
|  | **需增加的指标：** |  |  |  |  |  |  |

对于表2，若您还有其他意见，请在这里填写：

表3 三级指标咨询表

| Second-level indicators | Third-level indicators | 很重要 | 较重要 | 一般  重要 | 不太重要 | 不重要 | 修改意见 |
| --- | --- | --- | --- | --- | --- | --- | --- |
|  |  | 5 | 4 | 3 | 2 | 1 |  |
| I-1人文关怀照护系统 | I-1-1养老人文关怀照护质量管理小组组建情况 |  |  |  |  |  |  |
|  | I-1-2养老人文关怀照护制度和规范制定、落实与检查情况 |  |  |  |  |  |  |
|  | I-1-3人文关怀照护相关理论与技能培训方案制定、实施与记录情况 |  |  |  |  |  |  |
|  | I-1-4人文关怀照护相关理论与技能考核与持续改进情况 |  |  |  |  |  |  |
|  | **需增加的指标：** |  |  |  |  |  |  |
| I-2养老护理人力资源 | I-2-1养老护理人员与老人人数配比合理 |  |  |  |  |  |  |
|  | I-2-2养老护理人员的执业资质 |  |  |  |  |  |  |
|  | I-2-3养老护理人员遵守职业道德，具有良好的职业素养 |  |  |  |  |  |  |
|  | I-2-4培养养老护理人员职业认同感和荣誉感，鼓励个人职业生涯发展 |  |  |  |  |  |  |
|  | I-2-5机构人员合作互助，工作氛围融洽 |  |  |  |  |  |  |
|  | **需增加的指标：** |  |  |  |  |  |  |
| I-3老人及家属 | I-3-1建立个人及家庭信息库（如家庭背景、生活习惯等） |  |  |  |  |  |  |
|  | I-3-2定期评估老人及家属的关怀需求 |  |  |  |  |  |  |
|  | **需增加的指标：** |  |  |  |  |  |  |
| II-1关怀性环境 | II-1-1公共环境布置温馨和谐，敬老文化氛围浓厚 |  |  |  |  |  |  |
|  | II-1-2老人居室宽敞舒适，干净整洁 |  |  |  |  |  |  |
|  | II-1-3配置休闲娱乐活动场所和物品，定期清洗和消毒 |  |  |  |  |  |  |
|  | II-1-4标识导视系统完善，简洁易于辨认，如温馨提示标识、公共安全标识等 |  |  |  |  |  |  |
|  | II-1-5提供适老化的辅助器具和设施 |  |  |  |  |  |  |
|  | II-1-6自理区、半自理区、非自理区分区明显 |  |  |  |  |  |  |
|  | II-1-7公共环境布置温馨和谐，敬老文化氛围浓厚 |  |  |  |  |  |  |
|  | **需增加的指标：** |  |  |  |  |  |  |
| II-2满足日常生活照护需求 | II-2-1定期评估老人饮食和营养状况及需求 |  |  |  |  |  |  |
|  | II-2-2提供营养均衡、符合民族风俗习惯及食品安全要求、适宜老人食用的膳食 |  |  |  |  |  |  |
|  | II-2-3提供助餐服务，保障老人在就餐途中的安全 |  |  |  |  |  |  |
|  | II-2-4评估老人睡眠情况与需求，提供良好的睡眠环境 |  |  |  |  |  |  |
|  | II-2-5评估老人卫生清洁需求，协助完成个人卫生护理，如协助沐浴等 |  |  |  |  |  |  |
|  | **需增加的指标：** |  |  |  |  |  |  |
| II-3满足身体健康照护需求 | II-3-1评估老人肌力情况和活动能力，安排并协助开展康复训练 |  |  |  |  |  |  |
|  | II-3-2动态评估老人身体健康状况，提供助医服务，如急救、转诊服务、陪诊等 |  |  |  |  |  |  |
|  | II-3-3定期评估老人自理能力，动态调整照护等级 |  |  |  |  |  |  |
|  | II-3-4定期对老人开展养生保健、疾病康复等健康教育 |  |  |  |  |  |  |
|  | **需增加的指标：** |  |  |  |  |  |  |
| II-4满足精神文化照护需求 | II-4-1评估老人精神文化服务需求 |  |  |  |  |  |  |
|  | II-4-2开设老年教育相关理论课程 |  |  |  |  |  |  |
|  | II-4-3定期组织春游、秋游等户外休闲活动 |  |  |  |  |  |  |
|  | II-4-4定期举办绘画、唱歌、观看电影、下棋、做手工等活动 |  |  |  |  |  |  |
|  | II-4-5设置阅读室（角），提供书籍、报纸和杂志 |  |  |  |  |  |  |
|  | II-4-6鼓励和支持老人参加互助养老志愿服务，发挥余热 |  |  |  |  |  |  |
|  | II-4-7组织老人参加各项体育健身活动 |  |  |  |  |  |  |
|  | II-4-8开展活动时，为老人提供必要的安全防护措施 |  |  |  |  |  |  |
|  | **需增加的指标：** |  |  |  |  |  |  |
| II-5满足爱与归属照护需求 | II-5-1动态评估老人心理状况，重点关注新入住老人、临终老人等，发现异常及时与老人及家属沟通 |  |  |  |  |  |  |
|  | II-5-2根据需要为老人提供情绪疏导、心理咨询等心理慰藉服务 |  |  |  |  |  |  |
|  | II-5-3对老人和家属开展生死教育，帮助树立正确的生命观和死亡观 |  |  |  |  |  |  |
|  | II-5-4提供老年群体间互动交流机会，帮助建立友好和谐关系 |  |  |  |  |  |  |
|  | II-5-5帮助老人与家属保持联系，鼓励家属探望和参与照护老人 |  |  |  |  |  |  |
|  | II-5-6提供社会志愿服务，满足老人社会情感陪伴需求 |  |  |  |  |  |  |
|  | II-5-7积极开展社会宣传，吸引社会力量支持、参与养老服务 |  |  |  |  |  |  |
|  | **需增加的指标：** |  |  |  |  |  |  |
| II-6沟通交往 | II-6-1护理人员着装规范，举止得体 |  |  |  |  |  |  |
|  | II-6-2对老人称呼恰当，沟通交流符合服务规范要求 |  |  |  |  |  |  |
|  | II-6-3耐心倾听，及时回应 |  |  |  |  |  |  |
|  | II-6-4服务态度好，对老人一视同仁 |  |  |  |  |  |  |
|  | II-6-5关心出园老人，电话回访询问老人身体情况等 |  |  |  |  |  |  |
|  | II-6-6与老人及家属建立信任、关怀的关系 |  |  |  |  |  |  |
|  | **需增加的指标：** |  |  |  |  |  |  |
| II-7个性化关怀 | II-7-1鼓励老人表达自身想法、愿望和需求 |  |  |  |  |  |  |
|  | II-7-2综合考虑老人各方面因素，提供并协助开展个性化活动 |  |  |  |  |  |  |
|  | II-7-3根据老人的生活习惯、自理能力等情况，合理分配房间床铺 |  |  |  |  |  |  |
|  | II-7-4允许老人按照个人喜好装扮房间 |  |  |  |  |  |  |
|  | II-7-5给老人过生日，给予温暖的生日祝福 |  |  |  |  |  |  |
|  | **需增加的指标：** |  |  |  |  |  |  |
| II-8  尊重、鼓励 | II-8-1尊重老人文化习俗与宗教信仰 |  |  |  |  |  |  |
|  | II-8-2尊重老人对个人问题的自主决定权 |  |  |  |  |  |  |
|  | II-8-3尊重老人个性，尽量保留原有的生活习惯 |  |  |  |  |  |  |
|  | II-8-4尊重老人知情权，及时提供与老人相关的活动、护理和服务信息 |  |  |  |  |  |  |
|  | II-8-5避免低幼化对待老人，不使用幼稚的语言、给老人取昵称等 |  |  |  |  |  |  |
|  | II-8-6照护过程中保护老人的隐私 |  |  |  |  |  |  |
|  | II-8-7尽量为老人安排同性别护理人员 |  |  |  |  |  |  |
|  | II-8-8具备同理心，在提供服务时尊重老人的感受 |  |  |  |  |  |  |
|  | II-8-9鼓励老人自我关怀，自觉采取有利于健康的行为 |  |  |  |  |  |  |
|  | II-8-10鼓励老人参与护理决策 |  |  |  |  |  |  |
|  | II-8-11认可老人的表现和人生经历，给予信心与希望 |  |  |  |  |  |  |
|  | **需增加的指标：** |  |  |  |  |  |  |
| III-1老人及家属 | III-1-1老人及家属对机构人文关怀照护服务的满意度 |  |  |  |  |  |  |
|  | III-1-2老人的生活质量 |  |  |  |  |  |  |
|  | **需增加的指标：** |  |  |  |  |  |  |
| III-2养老护理人员 | III-2-1养老护理人员对工作的满意度情况 |  |  |  |  |  |  |
|  | III-2-2养老护理人员的职业认同及归属感 |  |  |  |  |  |  |
|  | **需增加的指标：** |  |  |  |  |  |  |
| III-3养老机构 | III-3-1机构管理者对人文关怀照护总体质量的自我评价 |  |  |  |  |  |  |
|  | III-3-2养老机构声誉和知名度的自我评价 |  |  |  |  |  |  |
|  | **需增加的指标：** |  |  |  |  |  |  |

对于表3，若您还有其他意见，请在这里填写：

第三部分 专家一般情况调研问卷

填表说明：本调查仅用作调查分析，且保证对您填写的信息内容予以保密。请您根据自身情况，在相应栏目中填写内容或打“√”，若有相关信息补充，请另做标记。

表1 专家基本情况调查表

| 姓名Name |  | 性别Gender |  | 年龄Age |  |
| --- | --- | --- | --- | --- | --- |
| 工作单位Workplace |  | | | | |
| 职称Professional title |  | 职务Position |  | 学历Educational level |  |
| 工作年限Years of professional experience |  | | 主要从事领域Primary field of practice |  | |
| 导师资格Supervisor qualification | □硕士生导师Master’s supervisor □博士生导师Doctoral supervisor □否None | | | | |
| 联系方式Contact information | 电话Telephone 邮箱Email address | | | | |
| 通讯地址Mailing address |  | | | | |

表2 您对咨询内容的熟悉程度调查表

| 熟悉程度[Degree of familiarity](https://dict.youdao.com/w/degree of familiarity/" \l "keyfrom=E2Ctranslation) | 非常熟悉(5)  Very familiar | 较熟悉(4)  relatively familiar | 一般熟悉(3)  moderately familiar | 不太熟悉(2)  not very familiar | 非常不熟悉(1)  not familiar at all |
| --- | --- | --- | --- | --- | --- |
| 专家自评Self evaluation |  |  |  |  |  |

表3 您对咨询内容的判断依据调查表

| 判断依据 | 专家自我评价 | | |
| --- | --- | --- | --- |
|  | 大Large | 中Medium | 小Small |
| 理论分析Theoretical analysis |  |  |  |
| 实践经验Practical experience |  |  |  |
| 国内外参考文献Refer to domestic and foreign information |  |  |  |
| 主观判断presentative judgment |  |  |  |

您对本研究的意见或建议：

本次函询到此结束，感谢您的支持，再次向您表示感谢！祝您幸福安康！

第二轮专家函询表

养老机构人文关怀照护质量评价指标体系的构建

专家函询（第二轮）

尊敬的专家：

您好！非常感谢您再次参加此次函询。我们是浙江省浙江中医药大学护理学院沈勤教授研究团队，现开展养老机构人文关怀照护质量评价指标体系的构建及初步应用的课题研究。该课题的第一轮专家咨询在您的悉心指导和大力帮助下已经完成，对此再次表示衷心的感谢！

根据第一轮咨询结果，并结合各位专家意见，经研究小组组内讨论，我们对各级指标进行了修订，二级指标具体修订如下：二级指标中将“满足日常生活照护需求”、“满足身体健康照护需求”、与“满足精神文化照护需求”合并为“满足基本生活照护需求”；修改“养老护理人力资源”为“护理人员队伍建设”，修改“沟通交往”为“沟通与安慰”，修改“尊重、鼓励”为“尊重与鼓励”，修改“个性化关怀”为“欣赏独特意义”，修改“养老护理人员评价”为“护理人员评价”，修改“III-1老年人及家属”为“老年人及家属评价”，并拆分为“老年人评价”“家属评价”；删除“I-3老人及家属”“III-3养老机构”，同时对相应的三级指标进行了修订。本轮咨询问卷共包括3项一级指标，11项二级指标和40项三级指标，现将第二轮专家函询表发给您，我们真诚期望继续得到您的指导和帮助！

请根据填表说明进行填写，恳请您**2周内**回复意见及建议。对于您的宝贵意见我们将严格保密。

最后，再次感谢您对本研究的支持，祝您身体健康，工作顺利！

第一部分 养老机构人文关怀照护质量评价指标

【填表说明】：

1.请您在表1、表2、表3中，根据各指标的重要性进行评分，其中：很重要（记为5分）；较重要（记为4分）；一般重要（记为3分）；不太重要（记为2分）；不重要（记为1分），请您在相应栏内打“√”。若需要修改或删除的内容请写在“修改意见”栏。若有新增的指标请写在“补充建议”栏。

2.请您在表2、表3判断各指标是否归属于相应分类，如“是”打“√”，如“否”打“×”。

3.请您填写专家基本信息登记表。

表1 一级指标函询表

| First-level | Definition | 重要性评分 | | | | | 修改意见 |
| --- | --- | --- | --- | --- | --- | --- | --- |
|  |  | 5 | 4 | 3 | 2 | 1 |  |
|  |  | 很重要 | 较重要 | 一般重要 | 不太重要 | 不重要 |  |
| I Structure | 构成人文关怀照护的基本条件和要素，如人员、培训情况、规章制度、硬件设备等。 |  |  |  |  |  |  |
| II Process | 护理人员根据老年人的状况及活动能力提供的服务活动及其他补充性活动。 |  |  |  |  |  |  |
| III Outcome | 人文关怀照护对老年人健康照护或结局的结果，也包括护理人员结局。 |  |  |  |  |  |  |
| 补充建议： |  | | | | | | |

表 2 二级指标函询表

| First-level indicators | Second-level indicators | 是否属于相应的指标类别 | 重要性评分 | | | | | 修改意见 |
| --- | --- | --- | --- | --- | --- | --- | --- | --- |
|  |  |  | 5 | 4 | 3 | 2 | 1 |  |
|  |  |  | 很重要 | 较重要 | 一般重要 | 不太重要 | 不重要 |  |
| I Structure | I-1人文关怀照护系统 |  |  |  |  |  |  |  |
|  | I-2护理人员队伍建设 |  |  |  |  |  |  |  |
|  | I-3关怀性环境 |  |  |  |  |  |  |  |
| 补充建议： |  |  |  |  |  |  |  |  |
| II Process | II-1沟通与安慰 |  |  |  |  |  |  |  |
|  | II-2尊重与鼓励 |  |  |  |  |  |  |  |
|  | II-3欣赏独特意义 |  |  |  |  |  |  |  |
|  | II-4满足基本生活照护需求 |  |  |  |  |  |  |  |
|  | II-5满足爱与归属照护需求 |  |  |  |  |  |  |  |
| 补充建议： |  |  |  |  |  |  |  |  |
| III Outcome | III-1老年人评价 |  |  |  |  |  |  |  |
|  | III-2家属评价 |  |  |  |  |  |  |  |
|  | III-3护理人员评价 |  |  |  |  |  |  |  |
| 补充建议： |  |  |  |  |  |  |  |  |

对于表2，若您还有其他意见，请在这里填写：

表3三级指标函询表

| 二级指标 | 三级指标 | 是否属于相应的指标类别 | 重要性评分 | | | | | 修改意见 |
| --- | --- | --- | --- | --- | --- | --- | --- | --- |
|  |  |  | 5 | 4 | 3 | 2 | 1 |  |
|  |  |  | 很重要 | 较重要 | 一般重要 | 不太重要 | 不重要 |  |
| I-1人文关怀照护系统 | I-1-1组建二级或三级人文关怀照护质量管理小组 |  |  |  |  |  |  |  |
|  | I-1-2制定人文关怀照护工作流程、工作规范和检查标准 |  |  |  |  |  |  |  |
|  | I-1-3建立正确的养老服务价值观和人文关怀服务理念 |  |  |  |  |  |  |  |
|  | I-1-4根据老年人能力评估等级及需求合理配置养老护理人员数量 |  |  |  |  |  |  |  |
|  | I-1-5建立志愿服务组织与管理体系 |  |  |  |  |  |  |  |
| I-2护理人员队伍建设 | I-2-1开展人文关怀照护知识与技能的培训 |  |  |  |  |  |  |  |
|  | I-2-2开展人文关怀照护知识与技能的考核与持续改进 |  |  |  |  |  |  |  |
|  | I-2-3开展关爱护理人员的相关活动 |  |  |  |  |  |  |  |
|  | I-2-4营造融洽互助的工作氛围 |  |  |  |  |  |  |  |
|  | I-2-5建立人文关怀照护激励机制，保障护理人员福利待遇 |  |  |  |  |  |  |  |
| I-3关怀性环境 | I-3-1居住环境温馨舒适 |  |  |  |  |  |  |  |
|  | I-3-2环境保障安全隐私 |  |  |  |  |  |  |  |
|  | I-3-3活动场所丰富多元 |  |  |  |  |  |  |  |
|  | I-3-4提供怀旧情感价值 |  |  |  |  |  |  |  |
| II-1沟通与安慰 | II-1-1沟通中注重人文关怀 |  |  |  |  |  |  |  |
|  | II-1-2评估老年人心理状况并提供心理慰藉 |  |  |  |  |  |  |  |
| II-2尊重与鼓励 | II-2-1尊重老年人的文化习俗与宗教信仰 |  |  |  |  |  |  |  |
|  | II-2-2尊重并保护老年人的躯体隐私及个人信息隐私 |  |  |  |  |  |  |  |
|  | II-2-3尊重老年人的人格尊严，避免低幼化对待认知正常的老年人 |  |  |  |  |  |  |  |
|  | II-2-4尊重老年人的知情权，提供照护、活动等相关服务信息 |  |  |  |  |  |  |  |
|  | II-2-5尊重老年人的监督权，鼓励共同参与机构管理 |  |  |  |  |  |  |  |
|  | II-2-6尊重老年人的个人意愿，鼓励共同制定照护计划 |  |  |  |  |  |  |  |
|  | II-2-7鼓励老年人自我关怀，重视自身健康并自觉采取健康行为 |  |  |  |  |  |  |  |
| II-3欣赏独特意义 | II-3-1关注老年人的生活背景及人生观、世界观、价值观 |  |  |  |  |  |  |  |
|  | II-3-2欣赏并肯定老年人的既往成就和现在表现 |  |  |  |  |  |  |  |
|  | II-3-3挖掘并发挥老年人的兴趣特长 |  |  |  |  |  |  |  |
| II-4满足基本生活照护需求 | II-4-1提供基础生活照料 |  |  |  |  |  |  |  |
|  | II-4-2落实安全保障措施 |  |  |  |  |  |  |  |
|  | II-4-3提供健康保健服务 |  |  |  |  |  |  |  |
|  | II-4-4提供文化娱乐活动 |  |  |  |  |  |  |  |
| II-5满足爱与归属照护需求 | II-5-1构建护理人员与老年人和谐人际关系 |  |  |  |  |  |  |  |
|  | II-5-2关注并协调老年人与老年人之间的人际关系 |  |  |  |  |  |  |  |
|  | II-5-3帮助老年人与家属保持联系 |  |  |  |  |  |  |  |
|  | II-5-4开展社会助老志愿服务 |  |  |  |  |  |  |  |
|  | II-5-5为临终老年人提供安宁疗护 |  |  |  |  |  |  |  |
| III-1老年人评价 | III-1-1老年人对人文关怀照护的满意度 |  |  |  |  |  |  |  |
|  | III-1-2老年人生活质量评价 |  |  |  |  |  |  |  |
| III-2家属评价 | III-2-1家属对人文关怀照护的满意度 |  |  |  |  |  |  |  |
| III-3护理人员评价 | III-3-1护理人员对人文关怀照护质量的自我评价 |  |  |  |  |  |  |  |
|  | III-3-2护理人员工作幸福指数 |  |  |  |  |  |  |  |

对于表3，若您还有其他意见，请在这里填写：

第二部分 专家一般情况调研问卷

填表说明：本调查仅用作调查分析，且保证对您填写的信息内容予以保密。请您根据自身情况，在相应栏目中填写内容或打“√”，若有相关信息补充，请另做标记。

表１专家基本情况调查表

| 姓名Name |  | 工作单Workplace |  |
| --- | --- | --- | --- |

表2 您对咨询内容的熟悉程度调查表

| 熟悉程度[Degree of familiarity](https://dict.youdao.com/w/degree of familiarity/" \l "keyfrom=E2Ctranslation) | 非常熟悉(5)  Very familiar | 较熟悉(4)  relatively familiar | 一般熟悉(3)  moderately familiar | 不太熟悉(2)  not very familiar | 非常不熟悉(1)  not familiar at all |
| --- | --- | --- | --- | --- | --- |
| 专家自评Self evaluation |  |  |  |  |  |

表3 您对咨询内容的判断依据调查表

| 判断依据 | 专家自我评价 | | |
| --- | --- | --- | --- |
|  | 大Large | 中Medium | 小Small |
| 理论分析Theoretical analysis |  |  |  |
| 实践经验Practical experience |  |  |  |
| 国内外参考文献Refer to domestic and foreign information |  |  |  |
| 主观判断presentative judgment |  |  |  |

本次函询到此结束，感谢您的支持，再次向您表示感谢！祝您幸福安康！
